# Valorisation of Biowastes for the Production of Green Materials Using Chemical Methods

**DOI:** 10.1007/s41061-017-0133-8

**Published:** 2017-04-03

**Authors:** Thomas I. J. Dugmore, James H. Clark, Julen Bustamante, Joseph A. Houghton, Avtar S. Matharu

**Affiliations:** Green Chemistry Centre of Excellence, University of York, York, North Yorkshire YO10 5DD UK

**Keywords:** Green chemistry, Biomass, Waste valorisation, Microwave

## Abstract

With crude oil reserves dwindling, the hunt for a sustainable alternative feedstock for fuels and materials for our society continues to expand. The biorefinery concept has enjoyed both a surge in popularity and also vocal opposition to the idea of diverting food-grade land and crops for this purpose. The idea of using the inevitable wastes arising from biomass processing, particularly farming and food production, is, therefore, gaining more attention as the feedstock for the biorefinery. For the three main components of biomass—carbohydrates, lipids, and proteins—there are long-established processes for using some of these by-products. However, the recent advances in chemical technologies are expanding both the feedstocks available for processing and the products that be obtained. Herein, this review presents some of the more recent developments in processing these molecules for green materials, as well as case studies that bring these technologies and materials together into final products for applied usage.

## Introduction

### Global Drivers

Sustainability is a much-used modern day buzzword that encompasses economic, social, and environmental values, i.e. the three pillars of sustainability. Sustainable development is development that meets the needs of the present without compromising the ability of future generations to meet their own needs [1]. However, the last one hundred years has seen global population multiply fourfold to 7.4 billion, global economic output increase more than 20-fold, and global material consumption increase eightfold. To date, about 72 billion metric tonnes (Gt) of materials are being consumed by humanity per annum and this number is projected to reach 100 Gt by 2030 [2].

Because of its abundance, ease of extraction and versatility, crude oil has been the primary feedstock for fuels and materials in modern day society for over a century [3]. Liquid fuels for the automotive industry is the most prolific use of crude oil; in the USA, fuels account for 76% of consumption as of 2014 [4]. However, many other materials are also derived from crude oil, most notably plastics (accounting for 6% of total oil consumption [5]), but pharmaceuticals, lubricants, adhesives, cosmetics, and food additives (and many others) are all typically manufactured from crude oil [6]. The manufacture of many of these products also requires the use of solvents, which are also largely crude-oil derived and account for ~1% of consumption [5, 7]. The outputs of chemical manufacturing are a success story reflected by the vast number articles and goods used in modern-day society that cater for daily well-being and lifestyle.

However, whilst crude oil may be the cornerstone of our established chemical industries it is a finite resource and its continued use represents a major environmental burden. Crude oil typically takes millions of years to form from biomass and migrate to reserves that are easy to tap [8–11], however as of 2013, our consumption is roughly 90,000 barrels per day (14.3 billion litres) [12]. Current estimates of remaining known reserves is between 900 and 1350 billion barrels [13]; although it should be noted that reporting figures do not always distinguish between proven reserves and probable reserves [14] and independent consensus from several reports has advised revising these figures downwards [15–18]. Crude oil consumption exceeded discovery in the late 1970s and on current consumption rates, crude oil is projected to run out approximately between 2080 and 2100 [13, 19]. Additionally, there are rising concerns about the environmental impact of extracting, refining, and using crude oil. Greenhouse gas (GHG) emissions are amongst the primary concerns; if we are to limit global warming to no more than 2 °C by 2020, then over 80% of coal, 50% of gas, and 30% of oil reserves are un-burnable [20]. Additionally the safety of the extraction/refining processes themselves are also of concern due to the environmental impact of oil spills [21]. Furthermore, increasing legislation, in particular REACH (Registration, Evaluation, Authorisation, and Restriction of Chemicals) in the European Union and its counterparts in other parts of world will certain raw materials’ availability, supply chains, and businesses [22].

In order to maintain worldwide quality of life and development levels, it is, therefore, imperative to access a clean, sustainable feedstock for our chemicals, materials, and energy. This led to the development of the biorefinery concept, whereby biomass, as opposed to crude oil, is the refinery feedstock and subsequently processed to separate the material into different fractions (such as biogas, sugars, proteins, oils, and cellulosic residues) before further processing into useful, marketable products and energy [23–27].

Whilst this concept enjoyed an initial surge in popularity in the mid-2000s, a number of problems soon arose—most notably in the use of biofuels. As fuel accounts for the largest consumption of crude oil, it is perhaps inevitable that biofuels were one of the first and fastest-developing bio-products to emerge [28–30]. The two most prominent biofuel processes at the time were fermentation to produce bio-ethanol to replace petroleum [25] and transesterification of vegetable oils with methanol to produce fatty acid methyl ester (FAME) biodiesel to replace mineral diesel [25, 28, 30–33].

The biggest problem that arose was the objection to using food-grade crops to produce fuels with malnutrition still being a global issue in what become popularly known as the “food vs. fuel debate” [34]. However, with biodiesel in particular, a number of operational problems with the fuels themselves started to become apparent. Vehicles, in particular, equipped with diesel particulate filters observed reduced engine performance over time due to injector clogging [35–37] and lubricant fouling [37–41] amongst others. This became largely attributed to polyunsaturated fats polymerising at higher temperatures and forming reactive oxygen radicals at moderate temperatures which in turn attacked the engine lubricant [40, 42]. Meanwhile, saturated fats, due to their higher melting points, solidify at temperatures unsuitable to the majority of European and North American environments, particularly during winter [43, 44].

Meanwhile, our current global society is suffering from escalating waste problems, which is becoming increasingly important and alarming in less developed and developing countries such as the BRICS (Brazil, Russia, India, China, and South Africa) and MINT (Mexico, Indonesia, Nigeria, and Turkey) nations, as well as in developed countries. The UN’s new sustainable development agenda sets key targets to reduce waste and also to protect natural resources, which are to be achieved by 2030 [45]:

- To manage and use natural resources sustainably and efficiently;
- To halve per capita global food waste at the retail and consumer levels and reduce food losses along production and supply chains, including post-harvest losses;
- To reduce waste generation substantially through prevention, reduction, recycling, and reuse;
- To ensure that people everywhere have the relevant information and awareness for sustainable development and lifestyles in harmony with nature;
- To support developing countries to strengthen their scientific and technological capacity to move towards more sustainable patterns of consumption and production.

To be truly sustainable, a more holistic approach to the biorefinery concept is required. As well as being renewable, an ideal feedstock also needs to be available on the timescales and volumes required for production. The means of processing it to a product should also be environmentally benign where possible, whilst ensuring that the product is still fit for purpose. The end of life of the product, as well as any waste generated during the process also requires attention. The now-famous 12 Principles of Green Chemistry developed by Anastas and Warner [46] in 1998 provided a good set of guidelines of how to produce chemical products and materials in an environmentally benign fashion. However, legislation is also coming in fast in other areas related to supply of feedstocks and handling of waste—most notably REACH (in the case of chemical feedstock for the former [22]) and the Waste Framework Directive (for the latter [47]) in Europe.

### Unavoidable Agri-Supply and Food-Supply Chain Wastes: An Interesting Renewable Resource

The twin problems faced by modern society of unsustainable dependence on non-renewable fossil resources (escalating demand with respect to supply) and escalating waste problems has the potential to be addressed by employing certain unavoidable wastes as raw materials. Unavoidable wastes such as agri- and food-supply chain wastes that arise from primary and secondary processing contain a wide range of highly functional molecules and are therefore prime candidates to be valuable raw materials for biorefineries for the generation of high-value products.^1^ Processes using renewable feedstocks are often closer to being carbon neutral compared with those of the conventional petrochemical routes [48, 49].

Even assuming that 100% of arable land is used for food production, the same efficiency is not the case for the crops themselves. Across the entire food and farming industries there are losses from harvest, through processing, all the way to retail, catering, and home consumption. This is in no small part due to inefficiency (spills, storage, etc.), but also nearly every crop has parts that are inedible to humans and therefore subsequently consigned to waste. For filter coffee production for instance, the pulp, and hull of the coffee beans are removed at harvest, during the roasting process the silverskins (or chaff) fall off, and finally, at the point of consumption boiling water is passed over the ground coffee to extract the flavour, leaving the grounds themselves behind as residues [50–52]. Whilst inedible to humans (and in the case of spent coffee grounds, to animals, notably ruminants [53] as well due to the presence of theobromine [50]), many of these residues still contain many functional materials and chemicals. For instance, relating to the food vs. fuel concept, spent coffee grounds also contain good amounts of oil (10–15% [50, 51])—Table 1 compares the oil yield of spent coffee grounds (SCG) to other typical oilseed crops. Several studies have successfully converted this oil to biodiesel which complies with both EN and ASTM standards [54–58] and London, UK, has now seen its first commercial SCG biodiesel plant open in 2014 [59].

**Table 1:** Typical oil content of various oilseed crops (wet basis)

Oilseed crop	Typical oil yield (%)
Spent coffee grounds	10–15
Chia	32–38 [60]
Corn	5–15 [61, 62]
Linseed	34–43 [63]
Olive	5–25 [64]
Rapeseed (Canola)	35–50 [65–67]
Soybean	10–20 [68, 69]
Sunflower	25–45 [70, 71]

However, even after the oil has been extracted, this still leaves the remaining residues behind, which account for >80% of the overall mass. SCGs have also been researched as solid fuels [56, 72, 73], but higher value applications for the solid portion to be researched have included: adsorbents for metal ions [74], capture of dyes [75, 76], and CO_2_ [77], extraction of antioxidants [78–83], as a growth medium for edible mushrooms [84] and fungal strains to release phenolic compounds [85].

Whilst, due to their high volumes, agri-residues have attracted a large amount of research interest, it is important to note that they are not the only source of biowastes. Forestry, for instance, also generates large amounts of wastes from tree felling in the form of smaller branches, leaves, needles, etc., which are removed before the larger tree logs are used for wood and paper [86]. Municipal maintenance and gardening also generate waste biomass in the form of pruning, grass cuttings, and hedge trimmings—generally considered (alongside household waste) as municipal solid waste (MSW) (sometimes termed organic fraction municipal solid waste, OFMSW) [87, 88]. Whilst composition varies, biomass still comprises largely the same types of major molecules (proteins, carbohydrates, and lipids) with lesser amounts of speciality molecules.

Both established and emerging global economies view waste as a bioresource for our next generation energy, chemicals, or platform molecules and materials, lessening the burden on crude oil, as of strategic importance. (Bio)waste as a resource has been recognised of national importance by the UK government following their 2015 report, “Building a high value bioeconomy: opportunities from waste” [89] as a result of the House of Lords Science and Technology Committee report, “Waste or resource? Stimulating a bioeconomy” [90] published a year earlier, both evoking the need for a UK bioeconomy for future sustainable development. The reports highlight a significant market for renewable chemicals, already estimated to be $57 billion worldwide and forecast to rise to $83 billion by 2018. Similarly, the United States Department of Agriculture (USDA) BioPreferred program reports that a bio-based economy contributes a total of $369 billion to the U.S. economy each year, while four million jobs were supported, directly and indirectly, by the bio-based economy [91]. Small and medium enterprises (SMEs) are an important driver for new growth as the EU bioeconomy (not restricted to waste feedstocks) has a turnover of about €2 trillion, employs around 22 million people, mainly in rural areas and often SMEs, and represents 9% of total employment in the EU. Each euro invested in EU-funded bioeconomy research and innovation is estimated to enable €10 of value added in bioeconomy sectors by 2025 [92].

Biorefineries will emerge alongside new infrastructure technologies. Some biorefineries will be standalone, others integrated with traditional petro-refineries, for maximum resource re-use. The best biorefineries will be those that are feedstock flexible, thus functioning all year round. New process intensification methodologies will need to developed in order to maximise resource and reduce waste as chemical manufacturing “does more and better with less”. Unavoidable food supply chain waste (UFSCW) represents an interesting “waste as a resource” option due to its high volume, chemical richness, and heterogeneity.

### High Value Applications

Whilst food vs. fuel dominated the early biofuels debate, it is important to note that the biorefinery concept extends far beyond fuels, both in terms of products to be produced, as well use of the entire feedstock. Examples of research beyond fuels into the solid portion of spent coffee grounds have already been given, but it is important to note that in the context of the biorefinery, for example, production of adsorbents for metal ions should be in addition to oil extraction. As with the petroleum industry, the feedstock should be processed to produce a range of products rather than honed in on one single product. As crude oil is the core feedstock for a range of materials besides just fuels, it is important that in order to succeed the oil refinery, the biorefinery should achieve the same goals. In fact, fuels from biomass should arguably be a last resort for two reasons.

The first is the concern about GHG emissions. Whilst biomass is renewable, as it is carbonaceous, burning it for energy still emits CO_2_ as part of the combustion process. As plants continually take in CO_2_ to produce energy and materials, the carbon emissions offset far more than for fossil fuels and are sometimes viewed as “theoretically” carbon neutral. However, once the full life cycle is taken into account beyond simply the carbon of the plant to include farming equipment, pesticides, conversion to fuel, etc., burning of biofuels still contributes a net increase in carbon emissions and can even be higher if, for instance, forests are cleared to grow biofuel crops [93–95].

The second is about efficient use of feedstocks. There exist a number of alternative ways to generate renewable energy such as solar, wind and hydroelectric. However, these sources cannot directly generate the carbon building blocks necessary to build the materials and products needed by society. As synthetic chemistry (whether for a single, pure product, polymer, or composite) essentially involves taking smaller, simple molecules and assembling them into larger, more complex ones, it is seen as wasteful to take biomass, which is already full of large, complex molecules and then burn it to reduce it back down to CO_2_—one of the simplest carbon-containing molecules. We should look to use the inherent structure and functionality provided by nature’s biopolymers and chemicals of life rather than trying re-synthesise from petroleum-based building blocks. Hence, making innovative use of currently low-value, underutilised biorenewable waste streams, especially unavoidable losses resulting from industrial practices (e.g. manufacturing, recycling) for the production of bio-derived chemicals, fuels, and other value-added functional materials is particularly important and attractive. Such waste valorisation practices also represent an imperative grand research challenge and a promising topic globally from both an environmental and economic point of view.

Herein, this review explores the major components of unavoidable terrestrial waste biomass, namely agri- and food waste. The first part will describe some of the advances in chemical technologies that allow processing of these components into higher value application materials. Secondly, some case studies where several aspects of these technologies are being brought together to help realise a “true” biorefinery where no part of the feedstock is lost to waste will be covered. Since polymers are the second largest product market after fuel, a significant amount of the materials and applications covered will fall into this category. This is to be a generic overview of some of the main processing means developed in the last few years and not intended to be exhaustive.

## Carbohydrates

**Fig. 1:**
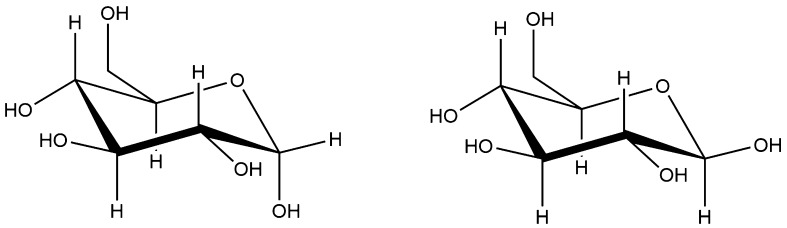
α- and β-glucose, the most abundant sugars

Of the three main biomass building blocks, carbohydrates are the most hydrophilic; as they are essentially hydrates of carbon, carbohydrates possess a hydroxyl group on every carbon atom to provide a high degree of polarity and hydrogen bonding. In biomass, carbohydrates mainly fall into two categories—simple sugars (monosaccharides) and dimers or polymers of those sugars (polysaccharides) with the remainder being derivatives of these. Of the simple sugars, the most abundant is glucose, which exists in two forms, α- and β-glucose, (structures shown in Fig. 1) with the only difference being the hydroxyl group at the 1′ position being either axial or equatorial. As glucose possesses six carbon atoms, it is classed as a hexose; the remaining hexose sugars are all analogous to glucose, again differentiated by the hydroxyl groups at the other positions being axial or equatorial. Other than hexose sugars, there are also pentose sugars which consist of five membered rings rather than six membered rings, again with the position of the hydroxyl groups determining the isomers [96].

### Sugars

**Fig. 2:**
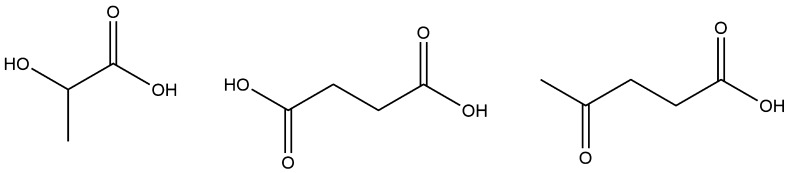
From *left* to *right*: lactic acid, succinic acid, levulinic acid

Since the simple sugars are so similar in structure, processing them using many “broad-brush” techniques is relatively easy. As sugars are also a primary energy source for many non-photosynthetic cells, fermentation to ethanol (for fuel or alcoholic beverages) has long been a means of converting sugars to other products [97–99]. As the process of fermentation involves the production of several intermediates [100], as do many other metabolic processes, many recent advances in sugar valorisation utilised fermentation to other target molecules, particularly platform molecules—those identified as being a set of basic chemical building blocks for synthesis into a wider range of molecules. Notable ones include lactic acid [101, 102], succinic acid, and levulinic acid [103]—structures shown in Fig. 2—although more will be covered throughout this review.

Lactic acid production via fermentation is an extremely well-known process, which was reported by Luedeking and Piret as early as 1959 using glucose as a feedstock and *Lactobacillus* bacteria [104]. Since then, through a combination of selective breeding of bacteria and enhancements of work-up and isolation techniques, the process has advanced considerably; a particular high point coming in 1996 with Linko and Javanainen reporting a yield of 98% from glucose using *Lactobacillus*—although several purification steps were still required to isolate the lactic acid from the broth [105]. As it possesses both an acid and alcohol moiety, lactic acid polymerises into polylactic acid (PLA) with relative ease. Polylactic acid is a well-established bio-based polymer, which is favoured for its biodegradability and compostability. It can, therefore, be used in many packaging and coating applications where biodegradability is desired, but is unsuitable for others—notably food packaging—due to its permeability and biocompatibility [106].

Succinic acid and levulinic acid are relatively more recent developments in the field (though receiving an increasing amount of attention). Originally, production of succinic acid (traditionally known as “spirit of amber”) was via the distillation of amber; it is also petrochemically produced from butane. It was identified by Zeikus in 1999 [107] for its potential to be produced from fermentation, albeit with low yields of around 45 g/L unless using specific bacteria from the *Succinogenes* species. It was identified in 2004 by the USDA as “one of the renewable building block chemicals with the greatest technical feasibility and commercial potential” [108], and the last few years has seen a surge in publications on its production. Levulinic acid was also mentioned as one of the 12 key platform molecules in the same report—it is traditionally produced from heating sugars in the presence of dilute acid [109]. Levulinic acid, therefore, has the longest history of bio-derivation, the key green chemistry driver for a switch to enzymatic production, in this instance to remove the mineral acid waste stream left over; to this end acidic ionic liquids have also been investigated [110].

**Fig. 3:**
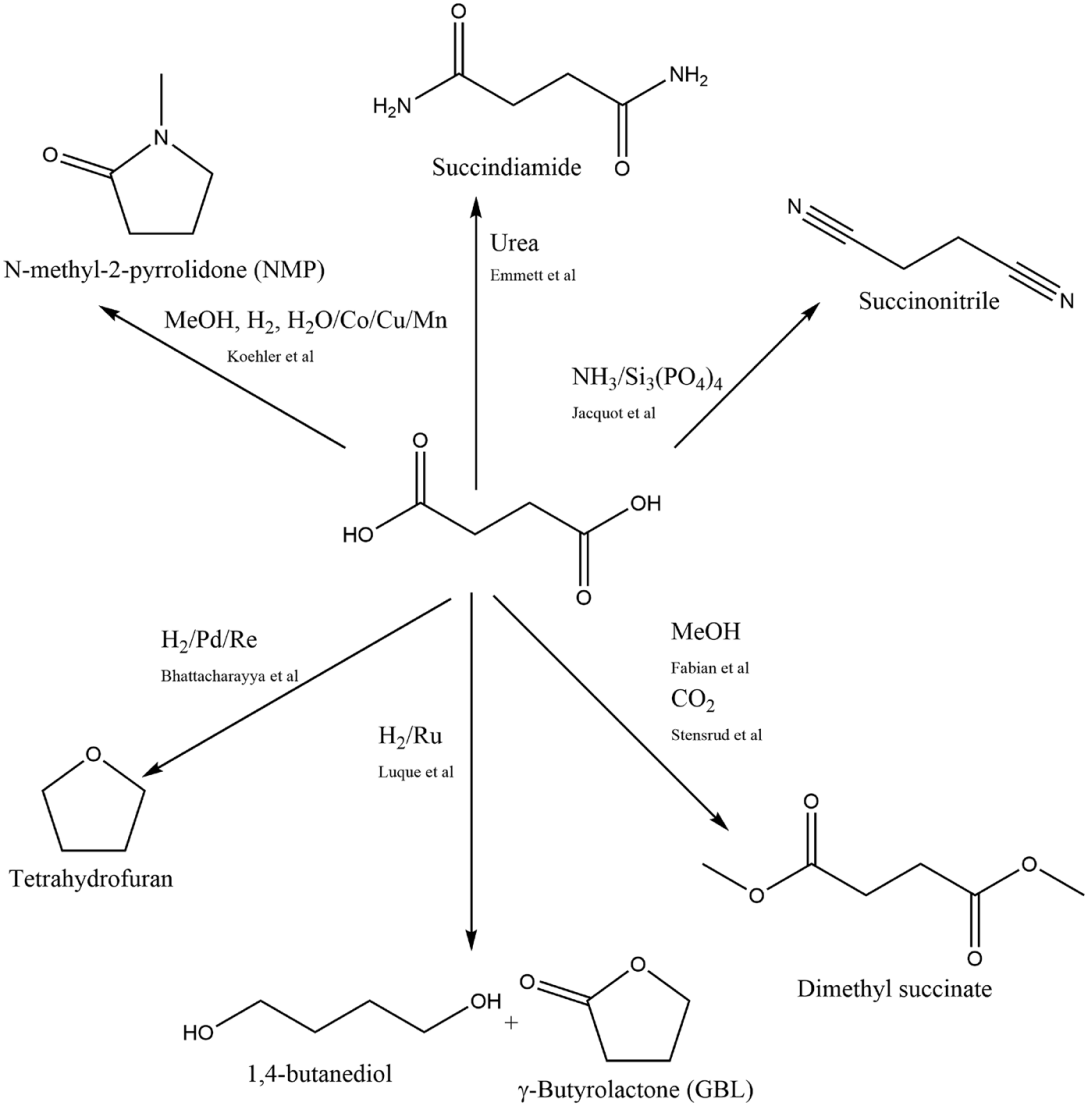
The chemical structure of succinic acid and a selection of conversions that can be performed on it [112–121]

Succinic acid is a popular choice as a platform molecule as just a few chemical steps allows conversion into a range of different molecules; Fig. 3 provides a (non-exhaustive selection). These include:

- Common manufacturing solvents, such as tetrahydrofuran and *N*-methyl pyrrolidone (NMP), although the latter is becoming strongly discouraged through REACH);
- Succinonitrile, a widely used electrolyte for Li-ion batteries (commonly produced by reacting toxic hydrogen cyanide with acrylonitrile [111]);
- Monomers for plastic production, such as 1,4-butanediol and succindiamide—although it is a di-acid, succinic acid itself is a monomer for polymer production.

**Fig. 4:**
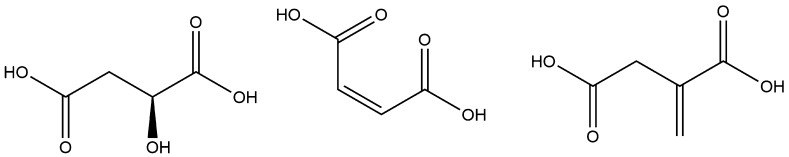
From *left* to *right*: malic acid, maleic acid, and itaconic acid

As a di-acid, polyesters are one of the most prominent polymer classes to which SA can be a monomer. In addition to SA, there are a many other di-acids that can be produced through fermentation including malic acid, maleic acid, and itaconic acid (structures shown in Fig. 4)—all of which can be converted to the subsequent diols.

Many of these molecules can be and are produced from petrochemical feedstocks (e.g. SA is produced by oxidation of butanediol, which is in turn produced by reacting ethyne with formaldehyde); therefore, in addition to bio-derivation, one of the advantages of using these platform molecules is that it avoids the use of toxic or otherwise harmful oxidation agents. Polyesters from combinations of these platform molecules have been successfully achieved in a number of studies using standard poly-esterification methods, such as with Ti(IV) tetra-*tert*-butoxide as a catalytic initiator [122] and through Michael additions using 1,3-dicarbonyls to produce a more complex branched network [123]. The resulting resins have had a range of properties reported, e.g. polydispersities from 2.8 to 85, molecular weight distribution from 8300 to 350,000 Da and glass transition temperatures from −51.2 to 135.4 °C—all of which further increased with Michael additions. Whilst the range of properties is promising, the authors did note two unwanted side reactions—isomerisation of itaconate units and saturation of C=C bonds—highlighting a need for good control measures in scale-up.

Whilst not typically produced chemically, polyhydroxyalkanoates (PHAs) are another class of linear polyesters worthy of mention—ones where the alcohol and acid moiety are on the same molecule. Many bacteria produce them as a means of carbon/energy storage from sugars and lipids. As previously noted, there are a wide range of different monomers, which can be produced from sugar fermentation there is similarly a wide range of polymers and resulting properties, which can be produced [124]. One of the most common is poly-(*R*)-3-hydroxybutyrate (P3HB), which typically employs glycerol as a feedstock; therefore, the lipids section will cover this in more detail.

One issue with the use of sugars for production of platform molecules is obtaining them from the respective biowastes. Solubilisation and extraction methodologies to extract sugars from complex matrices, such as pressing, ultra-filtration, and hot acid extraction have all been trialled [125, 126], but increasingly, free sugars in mixtures have also been studied for direct fermentation. Table 2 presents a range of substrates, bacteria, and yields of succinic acid and lactic acid reported from various studies. The results show many high (>70%) yields, indicating the potential for such a biorefinery concept to be adaptable to many different types of biowastes. It is worth noting that whilst lactic acid yields are notably generally superior to those from succinic acid, this is partly attributed to the fact that lactic acid fermentation is a much more established technology than succinic acid fermentation; however, using mixed waste feedstocks is still a comparatively recent development in the field. This suggests that as technology increases, or more efficient bacterial strains evolve, there is still very good scope for yields of succinic acid to increase over the next few years.

**Table 2:** A summary of some of the lactic acid and succinic acid yields from various food waste feedstocks

Feedstock	Bacteria	Yield (g/g)	Authors
Lactic acid			
Jackfruit seeds	*Streptococcus equinus*	0.62	Nair et al. [127]
Corn stover	*Lactobacillus pentosus* FL0421	0.66	Hu et al. [128]
Hydrolysed bakery waste	*Thermoanaerobacterium aotearoense LA1002*-*G40*	0.89	Yang et al. [129]
Sweet sorghum juice	*Bacillus coagulans*	0.92	Wang et al. [130]
Bakery waste	*Lactobacillus casei*	0.94	Kwan et al. [131]
Mixed food waste	*Lactobacillus casei*	0.94	Kwan et al. [131]
Succinic acid			
Cake	*Actinobacillus succinogenes*	0.25	Zhang et al. [132]
Pastry	*Actinobacillus succinogenes*	0.32	Zhang et al. [132]
Bread	*Actinobacillus succinogenes*	0.47	Zhang et al. [132]
Cane molasses	*Actinobacillus succinogenes*	0.55	Liu et al. [133]
Wood hydrolysate	*Mannheimia succiniciproducens MBEL55E*	0.56	Kim et al. [134]
Corn stalk	*Actinobacillus succinogenes*	0.66	Li et al. [135]
Cotton stalk	*Actinobacillus succinogenes*	1.23	Li et al. [135]

### Starch

**Fig. 5:**
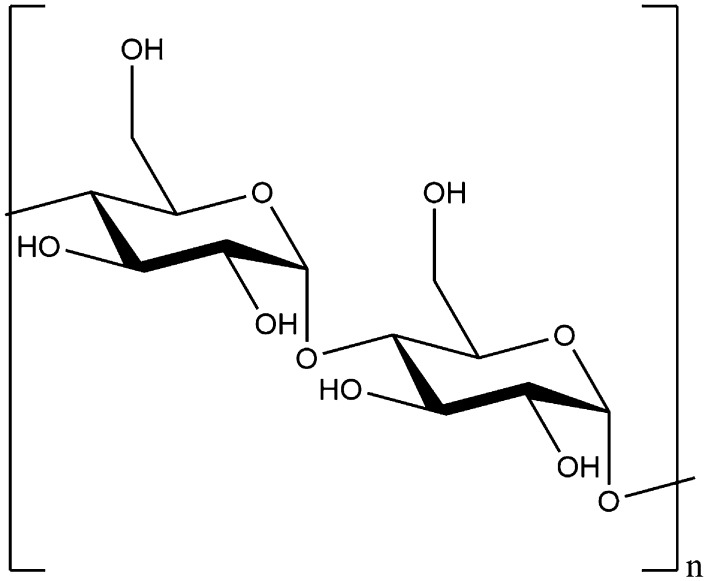
The generic chemical structure for starch

In addition to simple sugars, carbohydrates consist of a wide range of more complex polysaccharide molecules, with starch and cellulose the two most common. The main component of starch is simply long, linear chains of α-glucose polymerised through the 1 and 4 carbons—see Fig. 5.

There are several noted starch-rich food wastes. For instance, roughly 19% of the weight of a potato is starch [136]. Current global production of potatoes reached 360 million tonnes in 2013, with the UK alone producing 5.5 million tonnes [137].

Starch has been used for decades as a thickening agent, primarily for cooking, but also to produce gels for adhesion purposes. Most modern starch-based adhesives require some means of chemical modification (typically acid- or alkali- based) prior to their use, but have still generally been restricted by water-stability or inadequate mechanical properties for advanced applications. A more recent development by White et al. investigated the use of controlled thermolysis to achieve modification [138]. They used corn starch in water and heated above 100 °C under pressure (both thermal and microwave heating methods were used) to maintain water in its liquid form, also known as superheated or super-critical water [139]. Superheated water has lower polarity compared to liquid water under standard conditions and higher diffusivity; the effect on the starch is to cause the structure to swell, producing a highly porous network. Cooling the gel then “locks” the expanded network in place. The water is then “flushed out of” the matrix via solvent exchange in order to prevent collapse of the network. Acetylating the expanded starch gel with acetic or propionic anhydride then produces the final adhesive. This material is of interest as it expands the range of surfaces, notably metals, that can be bound using starch based adhesives [140]. However, the key property that differentiates this material from other adhesives is that is only exhibits adhesive properties within a certain temperature range, varying according to the binding surfaces [141]. This allows for selective bonding and de-bonding, therefore, enabling recycling at the end-of-life of the product. This has successfully been achieved in the production of carpet tiles [142]. At present, carpet tile production occurs by irreversibly binding the fabric layer to a bitumen base, leaving no option, but landfill at the end of life. By de-bonding via heating, the base is recovered for re-use with a different material, whilst the fibres can be recycled. This, therefore, represents a significant development for the production of green materials as it uses a renewable resource (starch), which is available in many waste streams from food production, prepares it without the use of harsh acids or alkalis, and enables the final product to be recyclable. Additionally, as starch is non-flammable, the resulting adhesive can produce the tiles without the addition of brominated flame-retardants that are required for many petroleum-based adhesives.

Another notable development for valorising starch has been the production of Starbon^®^ materials—mesoporous carbonaceous materials derived originally from starch, but now from a variety of polysaccharides. This process, developed by Budarin et al. [143–145], involves heating starch in water under pressure, in the same manner as producing the initial expanded starch gel for switchable adhesives. The material is then doped with a catalytic amount of organic acid (e.g. *p*-toluene sulfonic acid) and then pyrolysed under controlled heating conditions (again to prevent collapse of the network) to 300–800 °C depending on the desired properties. The higher the temperature, the more hydrophobic the surface properties [146–150]. This process has also been shown to work on alginic acid—another polysaccharide material obtained from seaweed.

These materials are finding use as replacements for activated carbons (AC)—carbon allotropes modified to provide high surface areas. ACs are widely used in applications such as water purification, filtration, flue gas scrubbing, and catalytic supports. Budarin et al. estimated that the current market for them is almost 1 Mt per year [144]. Because of the high porosity, particularly mesoporosity (2–50 nm pores [151]) generated through the gelation process, these new materials have frequently shown superior properties as adsorbents compared to their AC counterparts—a trait largely attributed to allowing adsorbates easier access to the inner micropores for adsorption. The model that increased surface area on activated carbon allows for greater adsorption to the surface is well established, but Hsieh et al. questioned how much of the surface area was actually available for adsorption. i.e. the increase in surface area was not worthwhile if it simply involved making up micropores too small for adsorbants to fit in, particularly for larger molecules. From subsequent Langmuir and Dubinin–Radushkevich modelling they suggested that increased mesoporosity would allow for increased diffusion throughout the material to access more of the AC surface [152].

For instance, Parker et al. tested the adsorption capacity of Starbons^®^ on a range of phenols [153]. S800 (starch derived and pyrolysed to 800 °C) with a surface area of 535 m^2^/g was shown to have an adsorption capacity for phenol of 87 mg/g. This compares to 37 mg/g with a coconut coir pith AC with surface area of 470 m^2^/g [154] representing a 2.35× increase in capacity with just a 1.13× increase in surface area. Apricot stone AC was shown to have a better adsorption capacity of 120 mg/g [155], but this required a surface area of 1306 m^2^/g; an improvement of just 1.38x despite a 2.44× increase in surface area. A800 (alginic acid derived and pyrolysed to 800 °C) performed even better, demonstrating an adsorption capacity of 89 mg/g despite a surface area of just 265 m^2^/g.

In a similar study, Garcia et al. tested the ability of Starbons^®^ to adsorb metal ions. Au, Pd, Pt, Ir, Ni, Cu, and Zn were all tested and successfully adsorbed by S800, with partial success in the adsorption of Au—an adsorption capacity of >3000 mg/g was reported compared to just 62–100 mg/g on AC [155]. Adsorption for the other metals were similar to AC results; however, in another crucial observation, both Parker and Garcia were able then to desorb the selected phenol and metal species, presenting the opportunity not just to remediate wastewater, but also to recover materials of interest. The recovery of the platinum group metals is of particular relevance due to their scarcity in the earth’s crust coupled with their increasing demand [156, 157]. A recent (2016) case study has been carried out by Tony et al. using Starbons to treat wastewater from commercial laundrettes [158].

The catalytic properties of these materials has also been successfully demonstrated. Because of their high adsorption capacity for metals, several studies exist using metal-based Starbon^®^ catalysts. For instance Luque et al. have reported the successful use of supported metal nanoparticles on Starbon^®^ for Rh, Ru, Pt, and Pd and their subsequent use in catalysing the hydrogenation of succinic acid and other platform molecules [114]. The team were able to achieve selectivity and conversions to their intended products of over 70% in less than 12 h with ruthenium being particularly effective—optimising reaction conditions led to over 90% selectivity and conversion for a range of organic acids [113]. Furthermore, they were able to tune their reaction conditions to achieve different target molecules. Other metal-centred catalytic studies have included Colmenares et al. work comparing the photocatalytic activity of TiO_2_ on Starbon against other AC materials. Over a threefold increase in the rate constants was reported in all instances [159]. Ojeda et al. have also reported their ability to produce Fe, Co, and Cu containing Starbon^®^ matrices via a similar method, though at the time of writing they have yet to test their activity on any reactions [160].

Several studies also demonstrate Starbons^®^ to be effective as solid acid catalysts. Many chemical transformations such as esterifications, hydrolysis, and hydration of alkenes rely on the addition of a catalytic amount of acid, usually sulfuric acid. After removal of the solvent, the added acid remains in solution and thus generates large amounts of acidic waste. Binding the sulphate group to an insoluble support (such as AC) allows the catalyst to be then filtered off and recovered after the reaction, thereby both enabling recycling and preventing the generation of harmful waste. After developing the materials, one of the first applications demonstrated by Budarin et al. was for sulphonated solid acid catalysis on esterifying succinic acid and ethanol. The Starbon^®^-based materials showed nearly a tenfold improvement on reaction rates compared to other solid acid catalysts and a twofold improvement over aqueous sulphuric acid [161, 162]. Other platform molecules such as itaconic acid and fumaric acid have demonstrated similar trends [163–165]. The material is also proving successful on other acid-catalysed transformations including alkylation and acetylation [166] and even for complex substrates such as the Ritter reaction [167]. It is unclear at present why the increased porosity improves catalytic performance, though a likely explanation is that it is to similar reasons for the adsorption properties; increased pore volume leads to more exposed acidic sites.

Whilst in early phases, the material has also been trialled as a stationary phase for HPLC where it successfully separated a mixture of carbohydrates [168].

### Cellulose

**Fig. 6:**
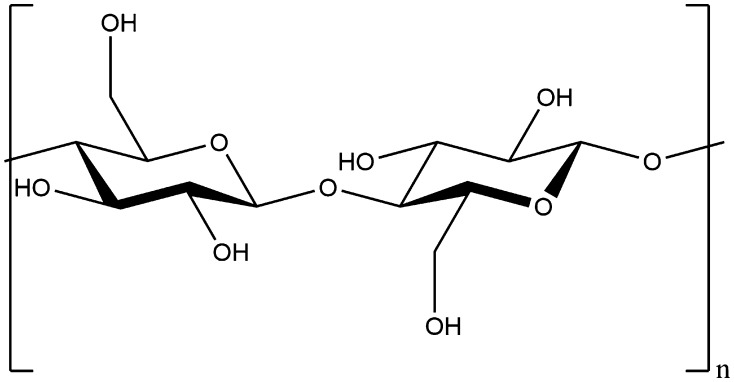
The generic chemical structure of cellulose

Cellulose, in a similar fashion to starch, is linked through β-glucose units at the 1 and 4 positions (see Fig. 6). Whilst the only difference between α- and β-glucose is that the hydroxyl group on the 1′ position isbeing axial and equatorial, respectively, this difference is key to very different resulting properties as it dramatically alters the bond angle between the two monomers and, by extension, the whole superstructure of the resulting polymer (as illustrated in Figs. 5, 6). Unlike starch, cellulose is insoluble in water, indigestible in many animals (including humans), and much more resistant to breakdown. For this reason, cellulose is a structural material in plants, rather than an energy store. Because it makes up the cell walls for the majority of plant cells, it is also the most abundant biopolymer on Earth.

Cellulose itself has a long history of applications in materials production, most notably as the raw material for paper and cardboard, as well as other materials such as cellophane and rayon. Since it is such a strong polymer, cellulose is very hard to break down without respective enzymes, which on one hand makes washing and recycling of the material relatively easy compared to some materials, but on the other can make it hard to process into other materials once recycling is no longer an option. In paper recycling for instance, the fibres get shortened in each recycling step, meaning after 5–6 stages the fibres are too short to be recycled into paper products anymore. One study addressing the valorisation of by-products from paper recycling came from Zhang et al. As with starch, cellulose also has a long history of usage in adhesive production. Zhang et al. investigated the microwave pyrolysis of waste paper and waste residues from the de-inking processes (e.g. fines and ink-sludges) used in paper recycling [169–171]. The standard products (depending on conditions) from microwave pyrolysis are biogas, biochar and a bio-oil, which itself comprises an aqueous and an organic fraction. When curing the organic fraction of the wastepaper bio-oil by hot-pressing between two aluminium plates, Zhang was able to achieve a maximum tensile strength of 2300 N for this adhesive—over twice as strong as the mandatory 900 N typically required for metal adhesion purposes. The de-inking residues did not fare as well, achieving only ~600 N under the same conditions; however, Zhang noted the high amount of sugars and aromatics in the bio-oil suggesting the potential for this material (or the precursor) to be a feedstock for other carbohydrate valorisation methods, particularly 5-hydroxymethyl furfural (HMF) and 5-chloromethyl furfural (CMF) production.

**Fig. 7:**
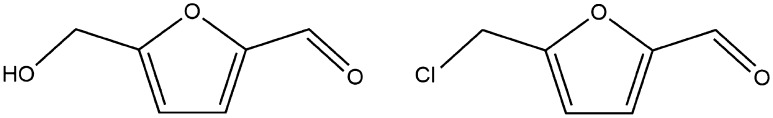
The chemical structures of 5-hydroxymethyl furfural (HMF-*left*) and 5-chloromethyl furfural (CMF-*right*)

In the context of green materials production from cellulosic biowastes, the production of the furan derivatives HMF and CMF (see Fig. 7) has also become a very interesting development.

**Fig. 8:**
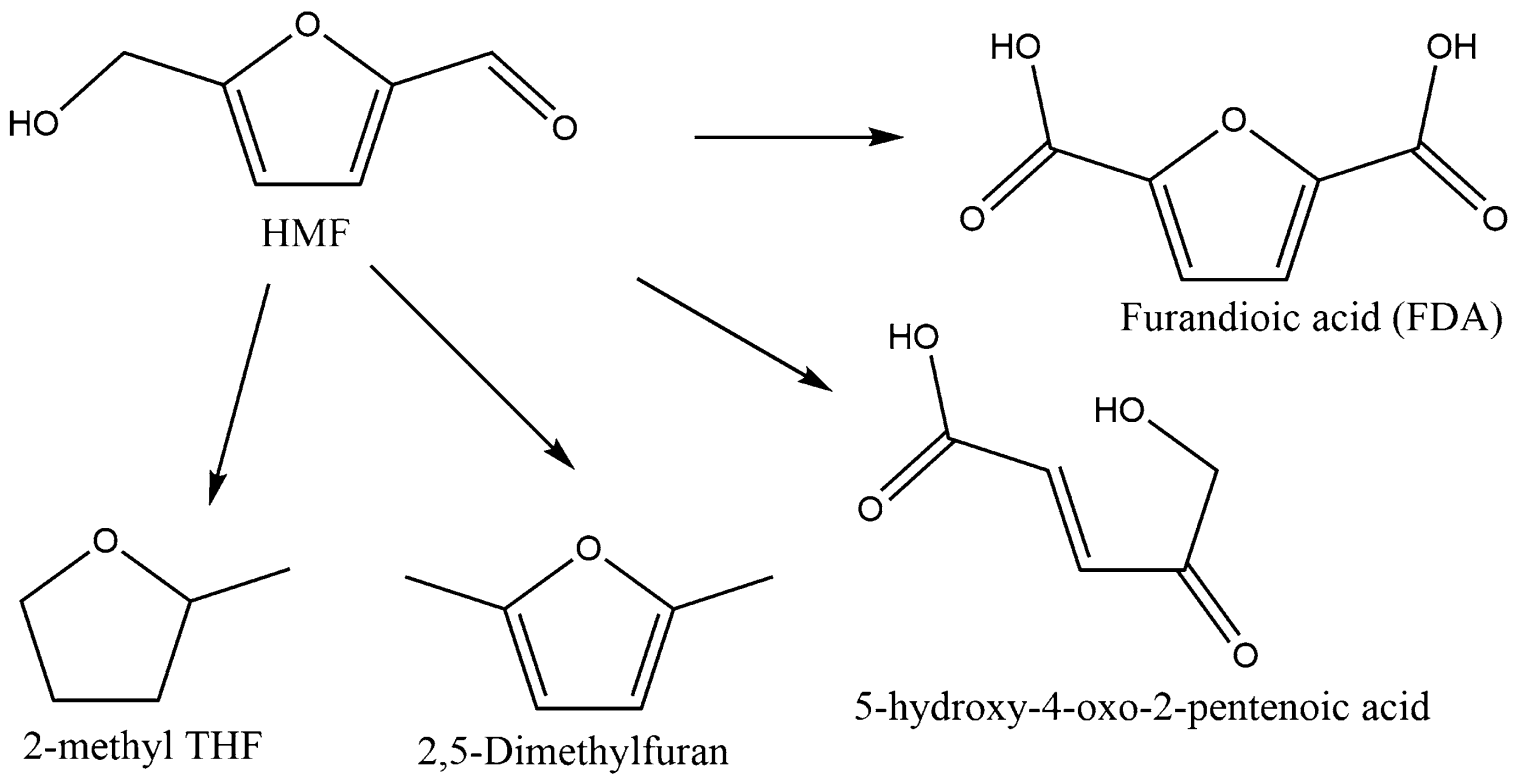
HMF, *top left*, and a selection of molecules it can subsequently be transformed to [175]

Many biomass sources naturally produce HMF through the degradation of sugars and is another noted platform molecule—Fig. 8 provides a selection of molecules derived from it through simple chemical syntheses. Again, the function of these molecules varies considerably. 2-methyl THF is seen as a potential candidate to replace THF as a solvent [172], 2,5-dimethylfuran (sometimes abbreviated to DMF, not to be confused with dimethyl formamide) is a biofuel [173], 5-hydroxy-4-keto-2-pentenoic acid possesses an acid and alcohol group, making a monomer for polymer production [174], as is furan-2,5-dioic acid (FDA) as a di-acid. One of the key challenges for the biorefinery concept is the relative lack of aromatics compared to petroleum. Lignin from woody-biomass represents the most abundant source of benzene-like rings; however, it is a non-uniform structure rendering it very difficult to break it down into uniform monomers or separate out bio-oils. HMF and CMF help address this challenge as they are, if not benzene-like, both based around aromatic rings.

**Fig. 9:**
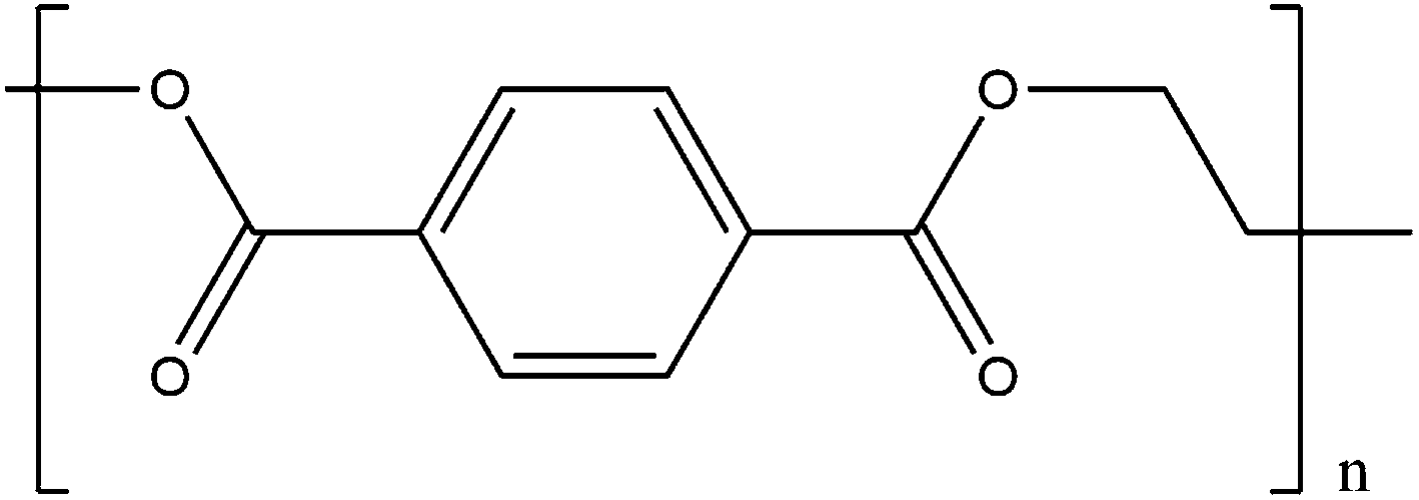
The chemical structure of PET

FDA especially makes for an interesting case study in replacing petroleum-based molecules. Polyethylene terephthalate (PET) is one of the most abundant plastics in circulation and is the most abundant of all polyesters. It has a range of uses, but is most widely recognised for food and drink storage, notably in drinks bottles. The two monomers used to make PET are ethylene glycol and terephthalic acid (benzene 1,4-dicarboxylic acid)—structure shown in Fig. 9. Even though it is furan-based rather than benzene-based, FDA is otherwise extremely similar in structure to terephthalic acid and can be substituted in for producing a new polymer, polyethylene furanoate (PEF) [174]. PEF has not only been shown to be successful in replacing PET in many applications [176], but is also now in commercial production for plastic bottles [177].

However, it is not only the applications of HMF and CMF that make it especially interesting from a biorefinery perspective, but the ease at which it can be manufactured from a range of cellulose based biowastes. HMF production is relatively simple to achieve by dehydrating sugars [178], but from polysaccharides it can be trickier because it has the ability to form polymers itself. Furfural (and furfural derivative) production from sugars has been in place since the late end of the nineteenth century using acid catalysis (both homo- and heterogeneous) to eliminate water [175, 179], with pentoses forming furfural and hexoses forming HMF. However, yields were low (~50%) with polymer coatings on the reaction vessels noted. However, the fact that these polymers partially prevented corrosion of the vessel wall by the acid catalysts employed partially mitigates that point. Since then, many other processes have emerged to improve the yield and efficiency, such as the Agrifurane, Suprayield and Westpro-modified Huaxia Tech processes. However, they all typically employ high (150–240 °C) temperatures, and where polymerisation is prevented, furfural breakdowns to a mixture of molecules (including formaldehyde) instead[180].

However, a new process developed by Mascal et al. demonstrates the ability to address both the aforementioned problems. By heating sugars in a mixture of aqueous hydrochloric acid and dichloromethane the sugars are easily dehydrated to the furan moiety, but the Cl^−^ groups from the acid replace the hydroxyl group at the 5-position, thereby preventing the polymerisation route [181]. The newly formed CMF is non-water miscible and, therefore, separates out into a biphasic system, decanted and able to undergo the same reactions as HMF [182, 183]. This process has been demonstrated to work on simple sugars, and cellulose and corn stover have also been successful, providing yields of >75% with levulinic acid as the only major by-product, as opposed to polymer resins or unwanted breakdown products. The fact that the by-product in this case is another valuable platform molecule, therefore, enhances both the green and economic credentials of the process. This process has also been successful under microwave conditions, presenting an opportunity to green the process further by reducing the time and energy inputs [184]. The one drawback at present, however, seems to be the inability to replace dichloromethane as the production solvent.

Neither starch nor cellulose typically exist in the ideal “pure” forms given in Figs. 4 and 5. The starch shown in Fig. 4 is the linear amylose variety, but there can also be branching at the 6-position to another α-glucose molecule, which is the amylopectin variety. Most starch consists of different ratios of the two, but amylase enzymes easily break down most forms and the ratios tend to make little difference to the described methods. Cellulose, on the other hand, frequently occurs entwined with large amounts of hemi-cellulose and pectin. Hemi-cellulose is a complex polysaccharide comprised of an indeterminate number and types of sugar molecules. As such, it is very hard to categorise and separate and many of the described valorisation techniques tend to focus on either removal of it for purified cellulose, or to break it down and ferment/process it along with the cellulose.

### Pectin

**Fig. 10:**
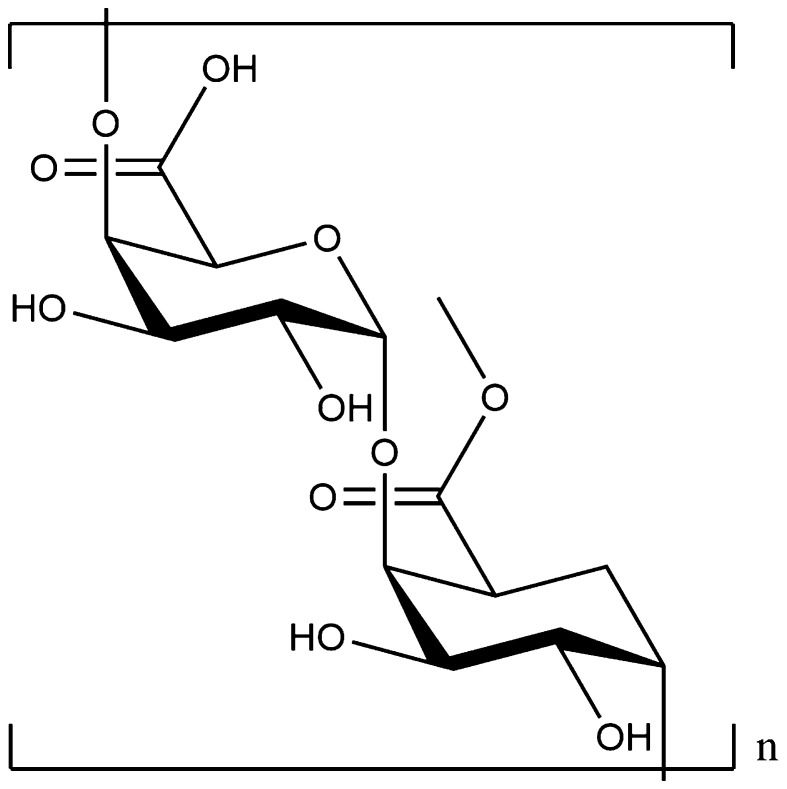
The general chemical structure of pectin

**Fig. 11:**
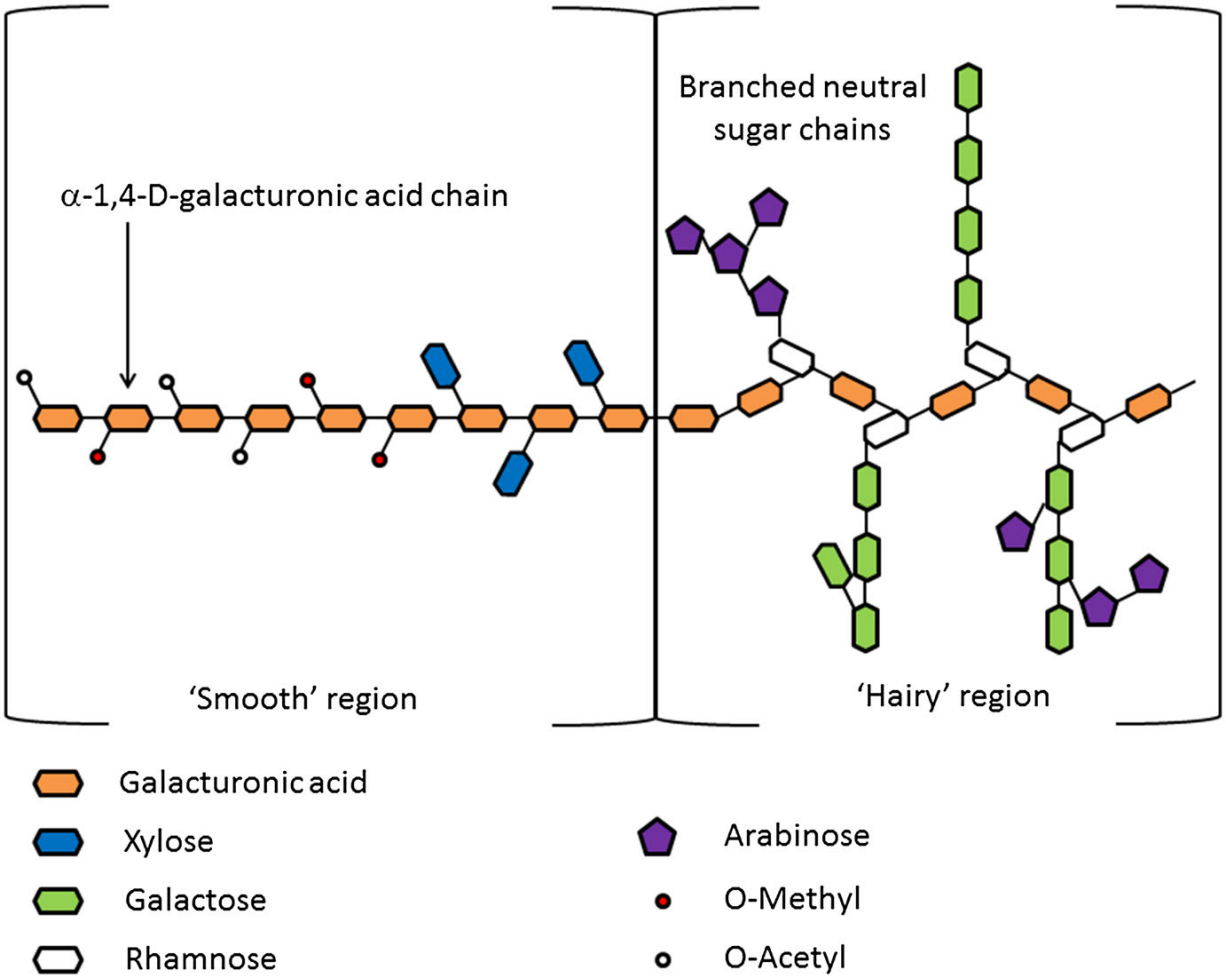
General structure of pectin [186]

Pectins, however, are also becoming of interest for material properties. They are another group of complex polysaccharides present in non-woody biomass mainly in the primary cell wall and intercellular regions [185]. They are composed of an α-(1-4)-d-galacturonic acid polymer chain (sometimes esterified with a methyl group—see Fig. 10) which, when unbranched, is known as homogalacturonan (HG) or the “smooth region”, and a “hairy” region, which comprises branched, neutral sugar chains (see Fig. 11).

The degree of esterification, level of branching, and, as with all polymers, average chain length and molecular weight distribution can all vary between biomass sources and can ultimately impact the overall properties [187]. Pectin extraction is a highly attractive valorisation method for fruit waste since pectin is present in high concentrations within fruit and has many applications within the food manufacturing industry for its ability to form a gel in water [188]. The degree of “smoothness” or “hairiness”, the degree of esterification (DE), amount of galacturonic acid (GA) and uronic acid, viscosity, and average molecular weight [189] are all properties governing the potential gelling properties of the pectin. This availability along with guaranteed demand means that pectin extraction could be one of the most lucrative aspects of fruit biorefinery systems. However, as these parameters differ depending on the source, if extraction of pectin for use in the food industry is the goal, rigorous analysis is needed to determine these values.

Mango waste, especially the peel, has been shown to have great promise as a potential source of food grade pectin with extraction yields of up to 21% [190] and promising values for the GA and DE. Traditionally, mineral acids have been employed in the extraction of pectins; however, this conflicts with the principles of green chemistry [191], due to their hazardous nature, and also they are unselective, meaning pectins extracted using this method have high neutral sugar content [192]. This technique is also time consuming [193], and although a high yield is generally obtained, the long extraction times at high temperature leads to thermal degradation [194, 195] of the pectin, lowering the average molecular mass [189]. More green extraction methods have been explored including using ammonium oxalate or ultrasound- and microwave-assisted extraction (MAE) techniques with quoted pectin yields of up to 11.6% [193, 196]. These extraction techniques have all been shown to be more selective and less harsh, yielding non-degraded pectin in high yields with good values for the GA and DE, along with high molecular mass and viscosity [189].

The variety of uses for sugars and starch is interesting as it highlights the potential to derive platform molecules from carbohydrate waste, analyse using HPLC, and convert them to materials all from carbohydrate-based resources.

## Lipids

**Fig. 12:**
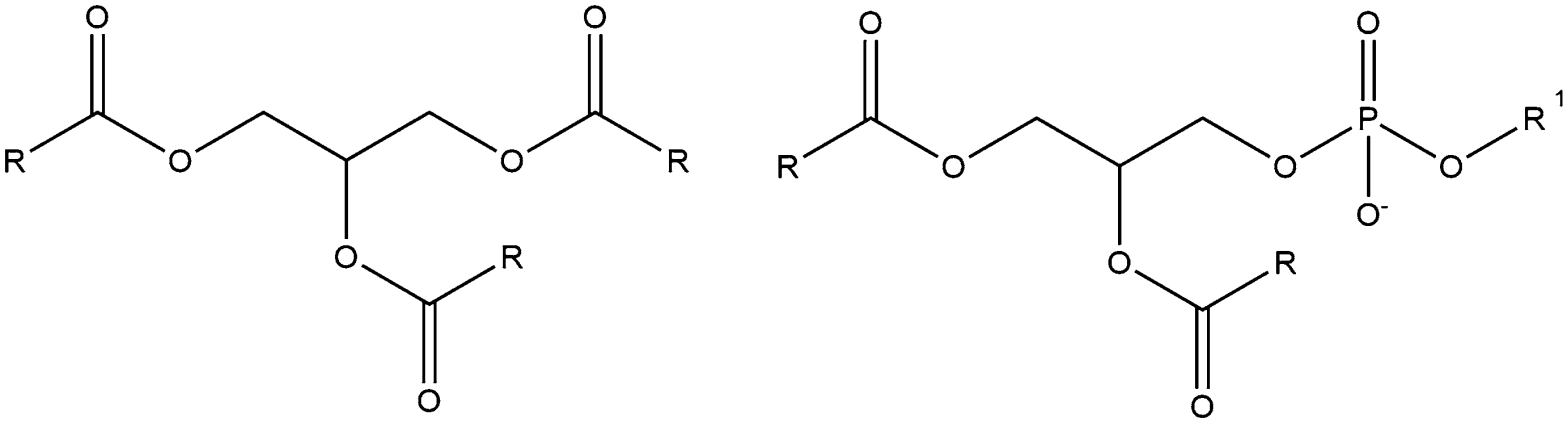
The generic structure of triglycerides and phospholipids, the two most common lipid systems

Converse to carbohydrates, lipids are the most hydrophobic of the three macronutrients and used in biological systems primarily for energy storage and as water repellents/surfactants. The two principal components in lipid systems are glycerides and fatty acids. Triglycerides, where three fatty acids are bound to one molecule of glycerol via ester bonds, occur more frequently in edible vegetable and animal oils and fats. Phospholipids, similar to triglycerides, but with one fatty acid replaced by a phosphate group, are the principal components of cell membranes, responsible for maintaining an aqueous environment inside the cell by creating a hydrophobic environment outside [197]. Figure 12 shows the structures for both.

**Fig. 13:**
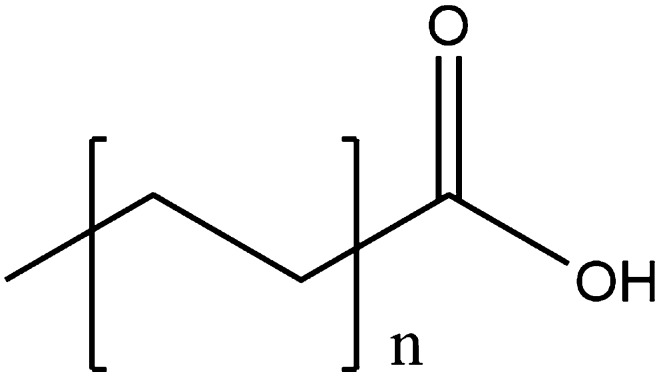
The generic structure of saturated fatty acids

**Fig. 14:**
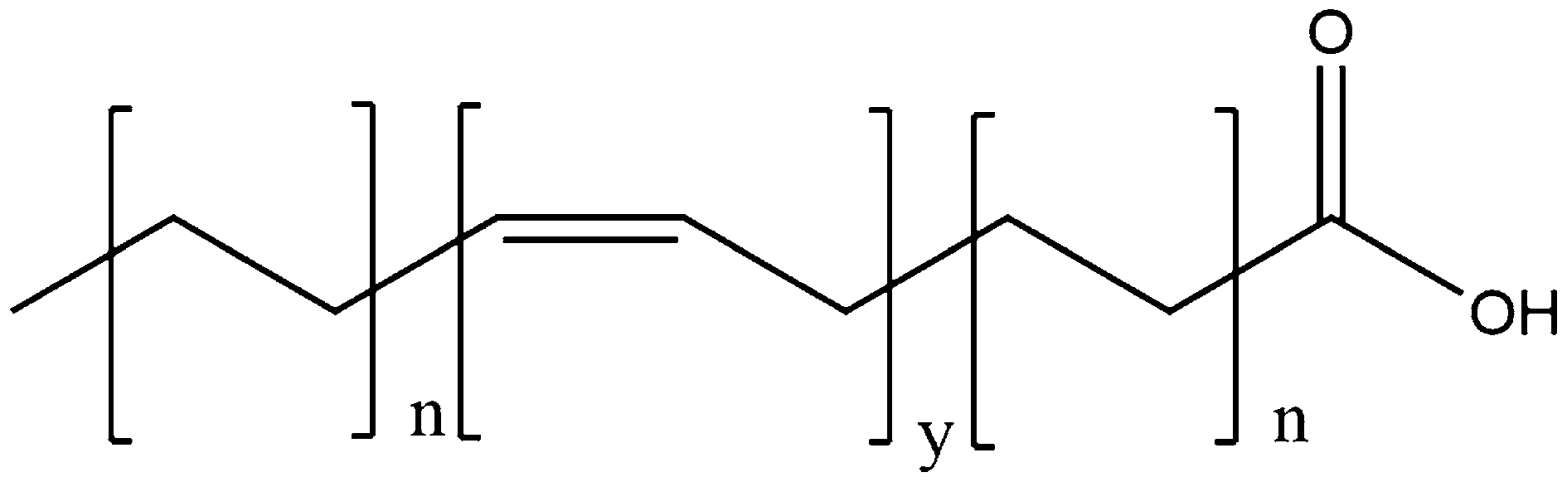
The generic structure of unsaturated fatty acids

Figures 13 and 14 give the generic structures for fatty acids. Saturated fatty acids are essentially simply long chain, unbranched carboxylic acids where, generally, *n* > 5. The most common ones are *n* = 8 or 9 (16 or 18 carbons long). Unsaturated (both mono- and polyunsaturated) fatty acids also consist of one or more double bonds along the chain, always separated by one carbon atom (i.e. no conjugation) and always *cis*.

Extraction of lipids from biomass also typically requires solvent extraction with non-polar solvents. Hexane is a common choice, but is on ChemSecs SIN list as of 2016 [198], substances of very high concern, under the criteria set up by REACH (the Registration, Evaluation, Authorisation, and Restriction of Chemicals) [22]. This being the case, heptane is a greener alternative (based on toxicity), which is becoming more attractive for this purpose due to similar solvation properties [185, 199, 200].

**Fig. 15:**
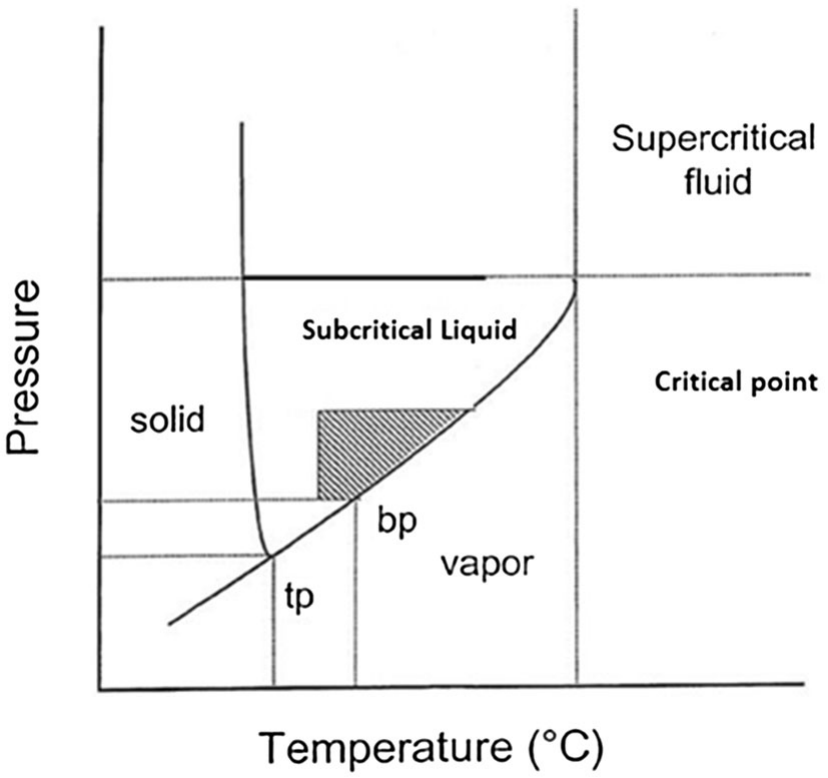
Typical phase diagram showing subcritical and supercritical conditions

Supercritical CO_2_ extraction is an interesting alternative green solvent to hexane. This is attractive since CO_2_ is low cost, inflammable, relatively inert, has low toxicity and a wide range of solvent properties depending on its temperature and pressure [200]. The term supercritical refers to a fluid, which is under pressure and temperature greater than the critical point (see Fig. 15) meaning that the fluid exhibits the properties of the gaseous and liquid phase whilst being a single phase [201]. The density of the fluid is closer to that of the liquid, and as solubility increases with density, pressure, and temperature, supercritical fluids exhibit a large absorption capacity. The gaseous properties of the super critical fluid allow for efficient extraction due to its highly diffuse nature. Once the extraction is complete the extract can be easily collected by reducing the pressure in the collection vessel until the fluid becomes a gas again, causing the extractant to “fall” out of the solution, negating the need for solvent removal through more conventional methods. Table 3 provides a selection of oil yields from supercritical CO_2_ from various feedstocks.

**Table 3:** A selection of oil yields from various feedstocks obtained from supercritical CO_2_ vs conventional solvent extraction (CSE) yield

Feedstock	Temperature (°C)	Pressure (bar)	Yield (%)	CSE yield (%)
Soybean [200]	50	552	18.3	19.0
Wheat straw [202]	100	400	1.8	1.17
Corn leaves [203]	65	400	1.76	
Linseed [204]	70	550	25	38
Rosehip [205]	40	300	8.78	8.99
Lavender [206]	48	90	4.9	4.9
Rice bran [207]	100	620	20.4	20.5

Another technique is use of subcritical water, previously mentioned in expanding starches. This allows for fast, cheap, green extraction [208] and low working temperatures [209]. Although the temperature is relatively high for water-based operations, the extraction method is so fast that it aids the retention of volatile and thermally sensitive components. This is where subcritical water extraction differs from classic steam distillation, because although steam distillation is run at 100 °C the run time is much longer, increasing the likelihood of compound degradation and loss of volatiles. Because the temperature of the water is increased, its polarity as a solvent decreases, allowing for extraction of compounds, which are not typically water soluble [210]. Subcritical water is generally cheaper than supercritical CO_2_ because increasing the temperature of a system is cheaper than increasing the pressure. Table 4 provides a selection of yields from subcritical water extraction from various feedstocks.

**Table 4:** A selection of yields from various feedstocks obtained from subcritical water vs steam distillation (SD) yield

Feedstock	Temperature (°C)	Pressure (bar)	Yield (%)	CSE/SD yield (%)
Ziziphora [209]	150	60	1.56	1.32 (SD)
Cretan Oregano [211]	150	60	3.76	3.58 (SD) 3.62 (CSE)
Rosemary [212]	150	20	0.05	0.05 (SD)
Kava [213]	100 175	60 60	9 10.4	5.7 (CSE)
Marjoram [214]	150	5	1.24	0.244

As one of the roles of lipids in the cell is to partition aqueous and non-aqueous environments, it is unsurprising that one primary means of valorising waste oils is as surfactants. The most common form is base hydrolysis to form fatty acid salts, which form the bulk material for soaps (hence, the common name for the reaction is “saponification”). However, the role of lipids as surfactants and emulsifying agents has attracted the attention of other fields as well. Do, Attaphong et al. studied the use of adding sulphate and phosphate groups to the acid head of free fatty acids to increase the hydrophilicity of the “head” end of the molecule. They were able to use them to make emulsions without the use of a co-oil with applications in cosmetics, vegetable oil extraction [215] (the latter is particularly relevant in light of the potentially upcoming restrictions on hexane) and viscosity modifiers for biofuels [216].

Another traditional use for animal and vegetable oils and fats prior to petroleum is as greases and lubricants to reduce friction between moving surfaces. Standards for performance in, e.g., engines have become more stringent since the advent of petroleum. In addition, there are many new applications, such as computer hard disks, that have emerged since the advent of petroleum, meaning that oils and fats cannot be directly applied to some traditional and novel applications, but require some form of processing first. Mobarak et al. investigated the long term stability (oxidative, hydrolytic, thermal, etc.) and properties of vegetable oils compared to their mineral counterparts [217]. They noted that vegetable oils were typically less thermally and oxidatively stable due to the presence of unsaturated fats. They also noted that they typically offered better lubricity during their lifespan, as well as lower volatility and, therefore, emissions. From this, they compared the different oil properties with industrial applications to suggest a “best-fit” for each. Since vegetable oils are major targets for automotive fuel in the form of biodiesel, it is perhaps unsurprising that the majority of literature on bio-lubricants have an emphasis on the same field—including the applications suggested by Mobarak. For instance, Bokade et al. studied the possibility of transesterification of vegetable oils to both biodiesel and bio-lubricants by altering the alcohol moiety [218]. They observed a lower conversion from triglycerides to monoesters as the alcohol chain length increased, but ultimately concluded that for *n*-octanol a 72% yield and 78% selectivity was still sufficient for the selected catalysts when considering the implications for being able to produce both commodities in the same process, same catalyst, etc. Salimon et al. attempted expand on this by using unsaturated fats to their advantage by adding other hydrophobic or hydrophilic groups to these sites and were, therefore, able to expand the usage of bio lubricants to other more advanced applications, notably as hydraulic systems and lightweight gas turbines [219].

**Fig. 16:**
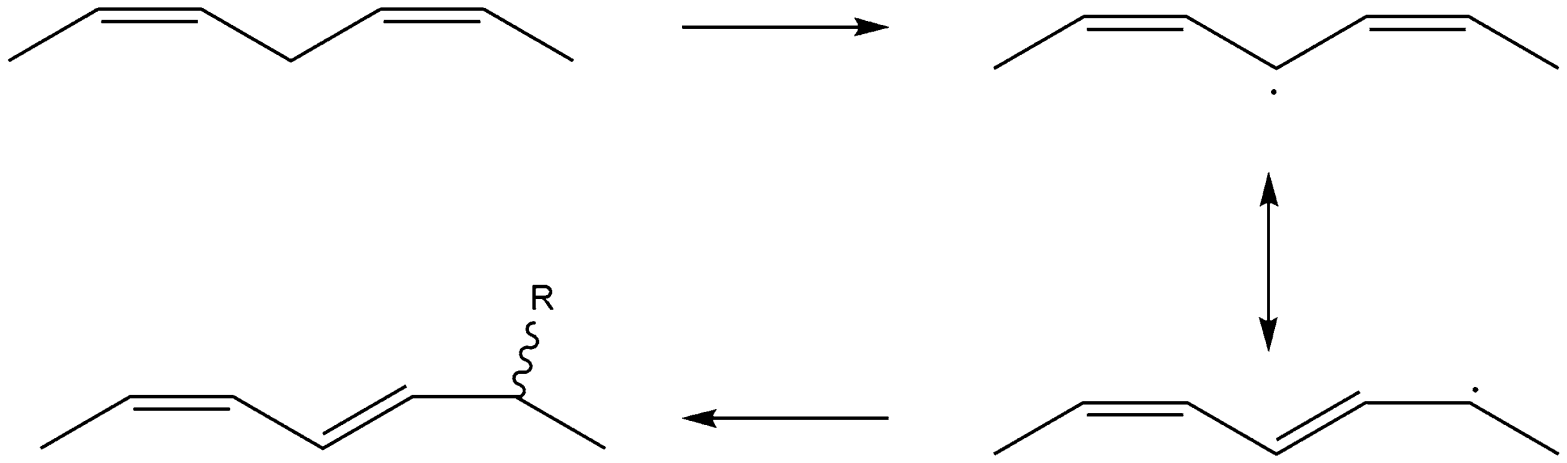
The migration of double bonds

Within the unsaturated fatty acids, the nature of the double bonds provides more options to perform chemistry on for higher value products. As with all double bonds, they are prone to autoxidation reactions [220]. However, in polyunsaturated fatty acids, as a carbon atom separates the double bonds, the double bonds are particularly prone to oxidation. This is because the process creates either an ion or radical intermediate that is stabilised by migration of one of the double bonds to form a conjugated system upon formation of the final product. Therefore, there is a strong thermodynamic driver due to the lowering the Bond Dissociation Energy of the allylic C–H bond [221–224] (mechanism shown in Fig. 16, where *R* is typically O_2_, RO_2_·, or RO·) [225].

**Fig. 17:**
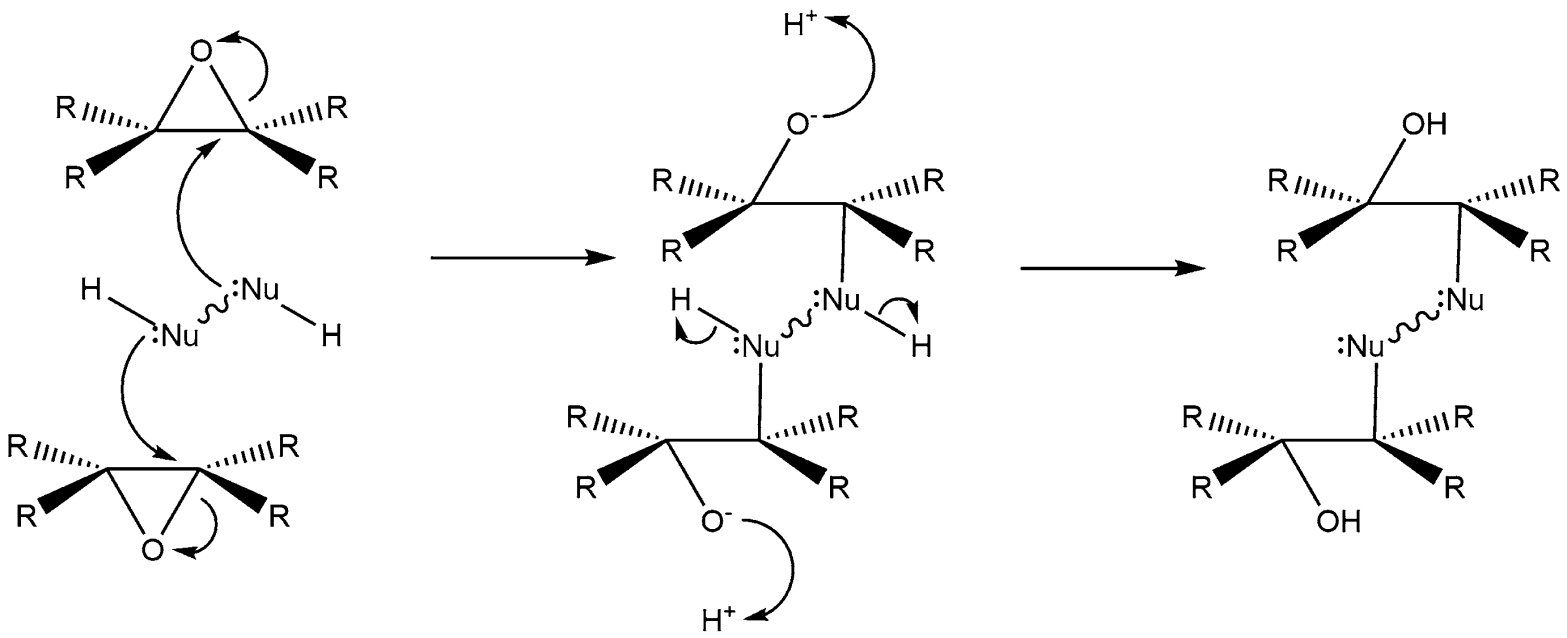
Cross-linking mechanism to form epoxy resins

Autoxidation of double bonds can also occur by direct addition across the double bonds to form epoxides [226–228]. The subsequent ring opening ability of epoxides from petroleum-based alkenes has long been exploited to create resins with a broad range of useful properties specific for application such as high strength, excellent corrosion and weather resistance, and excellent electrical insulation [229, 230]. Cross-linking agents are frequently used to open the epoxide rings and form a polymer matrix (structure shown in Fig. 17) [231].

**Fig. 18:**
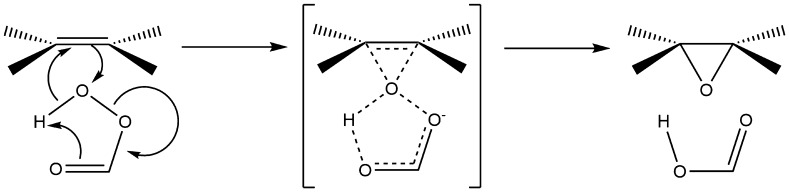
The Prilezhaev process for forming epoxides

However, the energy required for epoxidation is greater than the energy required for breaking the allylic C–H bond [232]; therefore, in addition, a petroleum-based alkenes the process to make traditional epoxy resins also deploys more specific oxidising agents and/or heat. For instance, Prilezhaev process uses formic acid and hydrogen peroxide to form performic acid, which is then used to carry out the epoxidation of the alkene [233, 234]—Fig. 18.

As fats and oils have always been commodities, epoxy resins from vegetables are also established products. However, the cross-linkers used are often still petroleum derived. Commonly, amines are used as hardeners due to the nucleophilic nature of the electron-dense nitrogen. Aliphatic amines are more nucleophilic than aromatic amines (where the aromaticity will withdraw electron density from the nitrogen), therefore, frequently involving the use of ammonia in preparation. Amine-based cross-linkers, therefore, potentially expose workers to harmful substances at both the production and usage stages of the operation through ammonia and amines, respectively. Park and Lin also voiced concerns about inadequate electrical and heat resistance [235].

Stemmelen et al. tried to use cysteamine hydrochloride, a dehydrated analogue of cysteine, to add directly to the double bonds of fatty acids through the thiol moiety before using the amine moiety to open the epoxides on another fatty acid chain as a means of creating a bio-derived cross-linker [236]. However, the initial reaction requires dioxane as a solvent, with chloroform, hexane, and diethyl ether as alternatives, all of which are typically derived from petroleum and face severe restrictions under REACH.

Gerbase et al. synthesised several epoxy resins from soybean oil using a range of petroleum and bio-derived acid anhydrides (including succinic anhydride and maleic anhydride) that were able to display a range of thermal and mechanical properties, demonstrating a good degree of versatility; however, they still required the use of a tertiary amine (often trimethylamine) as a reagent [237]. Mahendran et al. carried out similar work with linseed oil, achieving similar results, but managing to replace the amine reagent with an imidazole catalyst [238].

**Fig. 19:**
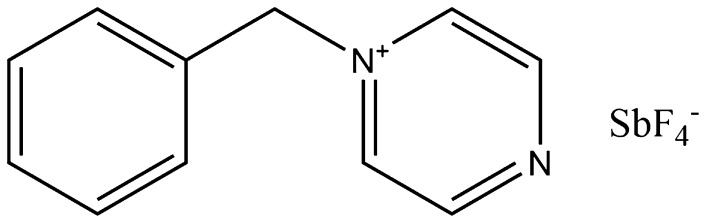
The chemical structure of *N*-benzylpyrazinium hexafluoroantimonate, BPH

In 2004, Park and Lin investigated the production of epoxy resins from soybean and castor oil without the use of a cross-linking agent, using only a catalyst, *N*-benzylpyrazinium hexafluoroantimonate (BPH—structure shown in Fig. 19) [239]. They reported that the resins both had a relatively low glass transition temperature, but a low coefficient of thermal expansion when compared to conventional diglycidyl ether of bisphenol A (DGEBA) resins synthesised under the same conditions [240, 241]. Similarly, in 2010, Altuna et al. synthesised pure epoxidised soybean oil (ESO) resins and DBEBA resins and mixtures of the two using a methyltetrahydrophthalic anhydride cross-linker and found that the glass transition temperature steadily decreased with an increasing amount of ESO. However, they also noted that the impact strength increased with a 40:60 mix of ESO:DGEBA, whilst the storage modulus remained relatively constant [242].

More recently in 2015, Ding et al. have reported using bio-based di-carboxylic acids as cross-linking agents for epoxidised linseed oil [243]. They observed that shorter acid chain length led to higher glass transition temperatures and better mechanical properties, but lower thermal stability.

The different properties resulting from the differing feedstocks and curing methods give the potential to produce a wide range of resins from lipids. Literature reviews on the subject seem to be largely positive, even if there are still issues to be resolved. Galià et al. [244] did an overview of vegetable oil-based polymers, highlighting deficiencies in flame retardant properties of pure vegetable oil epoxy resins and suggested that in order to obtain desired hardness properties, traditional cross-linkers used in petroleum resins would not be sufficient. They highlighted a need for novel resins to be developed. This was echoed by both Lu and Larock [245], who also noted that many of the cross-linkers needed for epoxy resins were still petroleum based. Tan and Chow [246] reported that epoxidised vegetable oil resins still needed to be blended with petroleum epoxy resins for many applications due to deficiencies in toughness and hardness. However, they also noted that advancements in oleochemical technology were addressing many of the shortcomings and concluded that “when being treated with proper curing agents, it is strongly believed that epoxidised vegetable oils have the potential to fully substitute current petroleum-based materials”. To this end, Ding and Matharu [247] conducted a review of the different curing agents available from biomass and also noted a variety of modified lipid-based curing agents available, as well as from carbohydrates and proteins, suggesting a great potential to address the concerns noted in the previous two reviews.

The production of such composites dates back to as early as 1997, where Crivello et al. explored a composite using glass-fibre mixed with linseed oil, which they suggested for use in standard domestic applications such as roofing, culverts, and low-pressure pipework [248]. More recently, in 2014, Supanchaiyamat et al. reported using epoxidised linseed oil and expanded starch (from the aforementioned Starbon^®^ process) to produce a fully bio-based composite with 227% improvement in tensile strength and 166% enhancement in Young’s modulus, compared to those with no added starch, suggesting it as a replacement for vinyl based films [249].

Other biopolymers from lipids have also been investigated. Polyurethanes, for instance, have been extensively studied by Petrovíc et al. by hydroxymethylating unsaturated acids—particularly oleic acids [250]. In his review, he noted the ability of ozonolysis of double bonds to produce diacids, aldehydes, and alcohols from double bonds for further polymerisation to polyurethanes and polyols [251]. He reported that the ability to select triglycerides with only one type of fatty acid allowed for greater control of mechanical properties, although noted that as all the major unsaturated fatty acids possess their first (counting from the acid moiety) double bond at the 9′ position, that ozonolysis or hydrolysis could achieve that required uniformity. However, he also cautioned that adding additional steps would inevitably drive up the cost of producing the material, which is undesirable for competing with cheap petroleum.

Overall, whilst there has been a large amount of research investigating the use of vegetable oils for bio-based polymers and resins, there is unfortunately no literature at the time of writing using these emerging technologies on waste oils such as used cooking oil or spent coffee oil. There has been research on applying these technologies to non-edible oils such as karanjia [252–254] and jatropha [255–257] with comparable results suggesting that the same technologies have the potential for application to waste oils—providing there is an appropriate amount of olefins to carry out the chemistry on. However, this is not confirmed at the time of writing, although Petrović did highlight the potential for fish oils to produce monomers via ozonolysis due to the high number of double bonds typically [251].

**Fig. 20:**
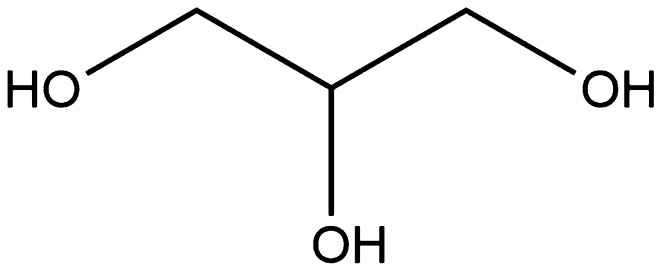
Glycerol. The major by-product from biodiesel and soap production

However, there has been a significant amount of work carried out on glycerol-based polymers, which is the primary waste product generated from soap production, biodiesel production, and any other process that cleaves the fatty acids from the triglycerides (structure shown in Fig. 20).

**Fig. 21:**
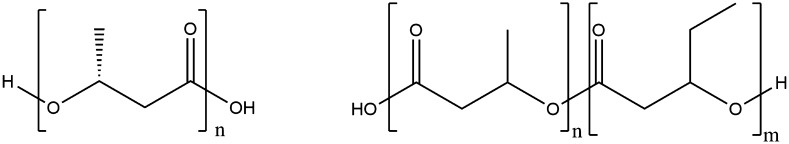
The chemical structure of poly 3-hydroxybutyrate (PHB-*left*), the most common class of polyhydroxyalkanoates, and its *co*-polymer poly (3-hydroxybutyrate-co-3-hydroxyvalerate) (PHBV-*right*)

A relatively well-researched area is fermentation to a class of polymers known as (PHAs), most notably poly 3-hydroxybutyrate (PHB—structure given in Fig. 21, left) [258, 259]—structure shown in Fig. 20. PHAs, as mentioned before (with levulinic acid given as an example) are a common class of biosynthesised polymers, but PHB is of particular interest. As well as being the most common of all PHAs, it is biodegradable and biocompatible leading to its application in medical devices and implants, in particular when produced with 3-hydroxypentanoic acid to produce poly 3-hydroxybutyrate-*co*-3-hydroxyvalerate (PHBV—structure given in Fig. 21, right), which is sold under the trade name Biopol.

Because of the biorefinery concept, it is perhaps inevitable that the production of these from crude glycerol (as opposed to pure, commercial grade) has attracted research interest. Mothes et al. investigated the effect of common contaminating salts from biodiesel production on the yield of PHB from glycerol and showed that whilst a 5% NaCl contamination led to a 48% reduction in PHB yield, K_2_SO_4_ showed minimal effect. Furthermore, the molecular weight distribution was between 620,000 and 750,000 g mol^−1^ was comparable to that of commercial PHB, indicating a good potential for crude glycerol mixes to be used in existing manufacturing methods [260]. Comparing commercial methods, Naranjo et al. demonstrated that the yield of PHB using glycerol as the feedstock could be as high as 62%. This is the same as for sugarcane bagasse and only 2% lower than whey, and they subsequently suggested that, since glycerol is produced as a waste product, the profit margin for PHB from glycerol could be up to 20% higher than glucose-derived PHB [261]. In a more complete biorefinery structure, Kachrimanidou et al. demonstrated not only the production of PHB from crude glycerol, but also the production of PHBV from a mixture of crude glycerol, sunflower meal (the leftover residue from oil extraction in biodiesel production) and levulinic acid, thereby using all by-products from biodiesel production [262].

Other monomers from crude glycerol have included the work of Papanikolau et al. in the production of 1,3-propanediol using crude glycerol (65% purity). This was obtained from a mixed-feedstock biodiesel production plant, with the same study also demonstrating the production of citric acid from the same feedstock—itself an important nutrient for fermentation of many of the products outlined in the carbohydrates section [263]. Vivek et al. built upon this to enhance the propanediol yield to as high as 0.83 g per g of glycerol [264]. Another example is the production of acrylonitrile from glycerol by Calvino-Casilda et al. by using niobium-doped Sb_*n*_V/Al_2_O_3_ catalysts [265].

## Proteins

**Fig. 22:**
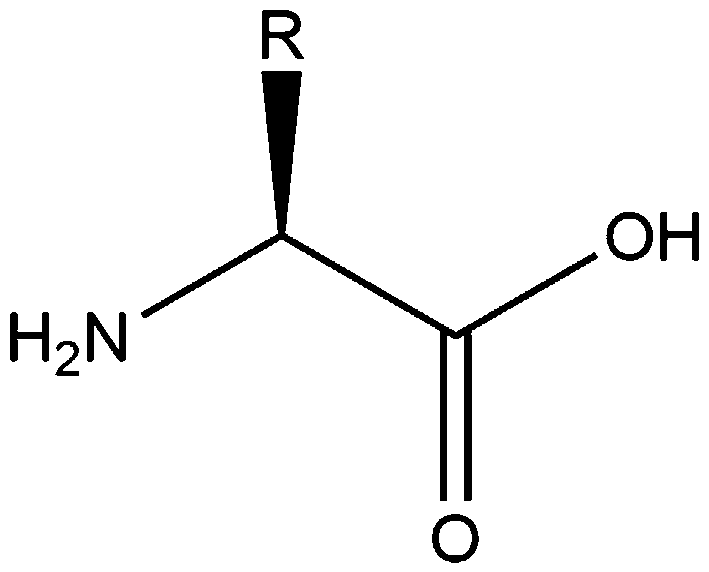
An amino acid, the building block for proteins

**Fig. 23:**
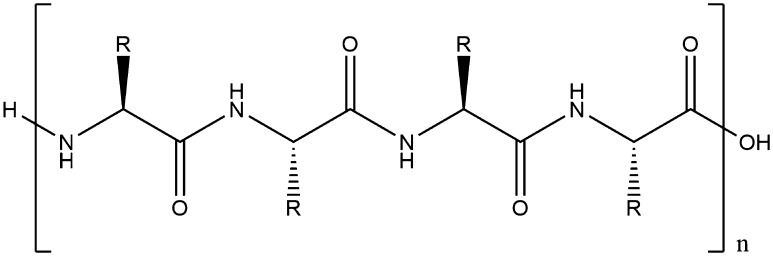
A chain of amino acids linked via peptide bonds

Of the three groups of macromolecules in biomass, proteins are arguably the most varied and complex. The main units of proteins are amino acids—essentially a carbon centre bonded to an amino group, a carboxylic acid group, a hydrogen atom, and an R group (see Fig. 22). The amino acids themselves join through amide bonds (more commonly known as peptide bonds in protein chemistry—see Fig. 23) between the amine and acid groups, but it is the R groups that are crucial for determining the nature of the protein. The R groups include other acids, amines, aliphatic chains, and aromatic rings providing a range of polarities and hydrophobicities. The sequence of the amino acids (primary structure) ultimately determines the (secondary and tertiary) structure and nature of the protein. In their review on isolation of vegetable proteins, Rodrigues et al. noted that “in theory, there is a limitless number of proteins with unique properties” [266].

Whilst the wide range of structures of proteins makes for a similar wide range of materials and products, they also make protein processing a very challenging task, as there is no “universal” approach. In addition, whilst rendering of fats and sugars for various purposes is established, protein extraction is a relatively new area. Nevertheless, there is still extensive research detailing the pros and cons of differing extraction and processing methodologies for different proteins, as well as their uses.

Most notably, as protein is a major macronutrient required by the body and traditionally consumed through meat, the rise in popularity of vegetarian and vegan diets has driven up the demand for plant-based proteins [267, 268]. Protease inhibitors from potatoes, for instance, are a very new development within the scope of potato valorisation. The protein obtained from potato is rich in lysine, which is one of the essential amino acids; because of this, potato protein is stated to be of higher quality than many other vegetable proteins [269, 270]. The protein quality is roughly 70% that of whole egg protein, as calculated using the EAAI (Essential Amino Acid Index) estimation of the amino acid composition [270, 271]. One interesting application for potato protein is because one of the protease inhibitors present (namely PI2) has been proven to be an appetite suppressant. It achieves this by inhibiting both trypsin and chemotrypsin, which constitute a negative feedback signal for cholecystokinin secretion, which creates a satiety feeling [269]. The study quoted that 82% of trypsin inhibition and 50% of chemotrypsin inhibition through potato protein is due to PI2. Kemin Health (marketing it under the trade name Slendesta^®^) has quoted that the required dose of PI2 needed to create the satiety effect is in the range of 300–600 mg and, therefore, can be taken in tablet form [272]. PI2 has a molecular weight of around 21 kDa and as such has scope to be separated via ultrafiltration; this system could theoretically be made to an industrial scale if PI2 separation was desired [273, 274].

A system has been developed that allows for PI2 concentration within the protein isolate; the separation of PI2 can be performed due to its relative stability concerning heat when compared to other potato proteins. PI2 is stable to above 70 °C, whereas patatin (another high concentration potato protein) is denatured and, therefore, precipitates out of solution at around 45–55 °C. This means that if the PFJ was heated to around 70 °C for a specified amount of time, the majority of the other proteins will precipitate out of solution, and the PJF could then be centrifuged to remove the precipitate and then the concentrated PI2 precipitated out using one of the different methods described above (ammonium sulphate, for example) [273, 274].

Within the field of chemistry, just as with carbohydrates and fats, another key use of proteins is the use of constituent amino acids as platform molecules. With the exception of glycine, where the R group is another hydrogen atom, all amino acids are chiral. However, unlike in traditional chemical synthesis, which produces both enantiomers in roughly equal amounts, in biomass the amino acids occur almost exclusively in the l-isomer. This makes them extremely useful in asymmetric synthesis either in introducing a chiral centre as part of the functional structure of the molecule or as a temporary attachment to create diastereomers and thus facilitate separation [275]. It should be noted, however, that the latter is frowned upon in terms of green chemistry, which seeks to avoid the use of temporary analogues, such as protecting groups—particularly if the unwanted enantiomer is then consigned to waste [46]. Nevertheless, asymmetric synthesis is an integral tool for synthetic chemists, particularly for drug development whereby one enantiomer may be inactive, or even harmful. The production of enantiomerically pure compounds similarly requires testing, purifying and quantifying in the production process. In order to interact differently with different enantiomers, many of the reagents and catalysts [276], HPLC stationary phases [277, 278], etc. often need to be in enantiomeric excess themselves, further highlighting the essential role of amino acids and proteins as platform molecules.

The field of health drug discovery has also highlighted other innovations for protein valorisation. Whey protein from cheese production is of particular interest in this field as it contains all 20 amino acids making it a very rich nutrient source for the body to construct its own proteins. Athletes and sportspeople will be familiar with the presence of whey protein powders in gyms and sports shops to enhance muscle growth [279–281], but the ability to help the body synthesise its own necessary proteins means it is also being investigated as an alternative to glucose as a drug-delivery media [282]. There has also been reported success in weight loss [283, 284], anti-cancer [285], tissue regenerating [286, 287], and anti-inflammatory medications [288]. Smithers has forecast a large growth in this field for whey protein; however, he noted the need to make use of emerging, non-thermal technologies, such as pulsed electric field [289], for extraction and isolation in order to make the applications cost-effective [290].

Collagen is another protein that has been studied for drug delivery purposes [291]. Collagen is the most abundant protein in animal bodies, providing the structural integrity for body tissue [292] and is a particularly abundant by-product from seafood production [293]. For applications, a drawback is that it is non-vegetarian friendly—although whey protein is similarly non-vegan friendly.

**Fig. 24:**
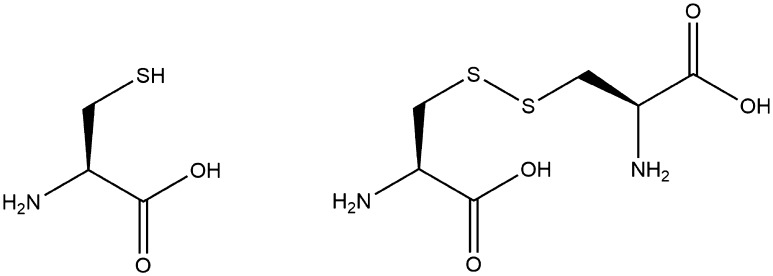
The chemical structure of cysteine (*left*) and cross-linking with itself in a disulphide bond to form cystine (*right*)

Keratin is another common polymer, which is of interest for its structural properties [294]. Keratin is an insoluble protein that makes up the exterior parts of animals such as hair, nails, claws, feathers, etc. Historically, the most prominent use of keratin has been the spinning of sheep’s wool or other hair/fur for material such as woollen garments or angora. However, the harder keratin types, such as those that make up feathers are now becoming more of interest due to the quantities of waste feathers generated in the poultry industry. The proteins here are typically hard, insoluble, and mechanically strong—properties generally attributed to the high amounts of cysteine side chains able to provide “built-in” cross-linking groups via disulphide bonds—see Fig. 24.

Tanabe et al. studied the polymerisation of films from keratin in a similar manner to resins from lipids [295]. However, as before, the use of a cross-linking agent was required to get the highest tensile strength of the films; however, glycerol proved to be the best cross-linking agent, whilst films mixed with chitosan also demonstrated superior mechanical properties to keratin alone. As both these materials are discarded as biowastes in large quantities, this presents an interesting opportunity for an integrated biorefinery by using feedstocks from different sources. However, from a green chemistry perspective, the use of sodium dodecyl sulphate and, particularly, 2-mercaptoethanol to extract and prepare a solution of keratin is less desirable. Poole et al. investigated the use of both chicken feather keratin and wheat gluten for the production of protein fibres, comparing them to other natural (e.g. cotton and silk) and synthetic (e.g. nylon and polypropylene) fibres [296]. Whilst on the low end of the molecular weight distribution range required, feather keratin still showed all the required properties in terms of crystallinity, cross-linking sites, and ability to form threads. Wheat gluten showed a more desirable molecular weight distribution, but did not perform as well in the rest of the properties desired. Furthermore, feather keratin estimates suggest them to be available in up to five million tonnes annually in very consistent quality, and unlike Tanabe’s work, did not require additional cross-linking agents. However, Poole et al. also noted the difficulty in solubilising the keratin that Tanabe had observed, suggesting the need for a green extraction method to be able to validate polymers and fibres from keratin as a “green” material.

## Case Studies

### Potatoes

This review so far has covered the different components of biomass and potential processing methods to different materials. In order to realise these concepts efficiently it is necessary to bring these methodologies together. This final section will look at some case studies, where (a) the different parts of a biowaste are refined into their respective materials, and (b) different materials are brought together to make finished products.

**Fig. 25:**
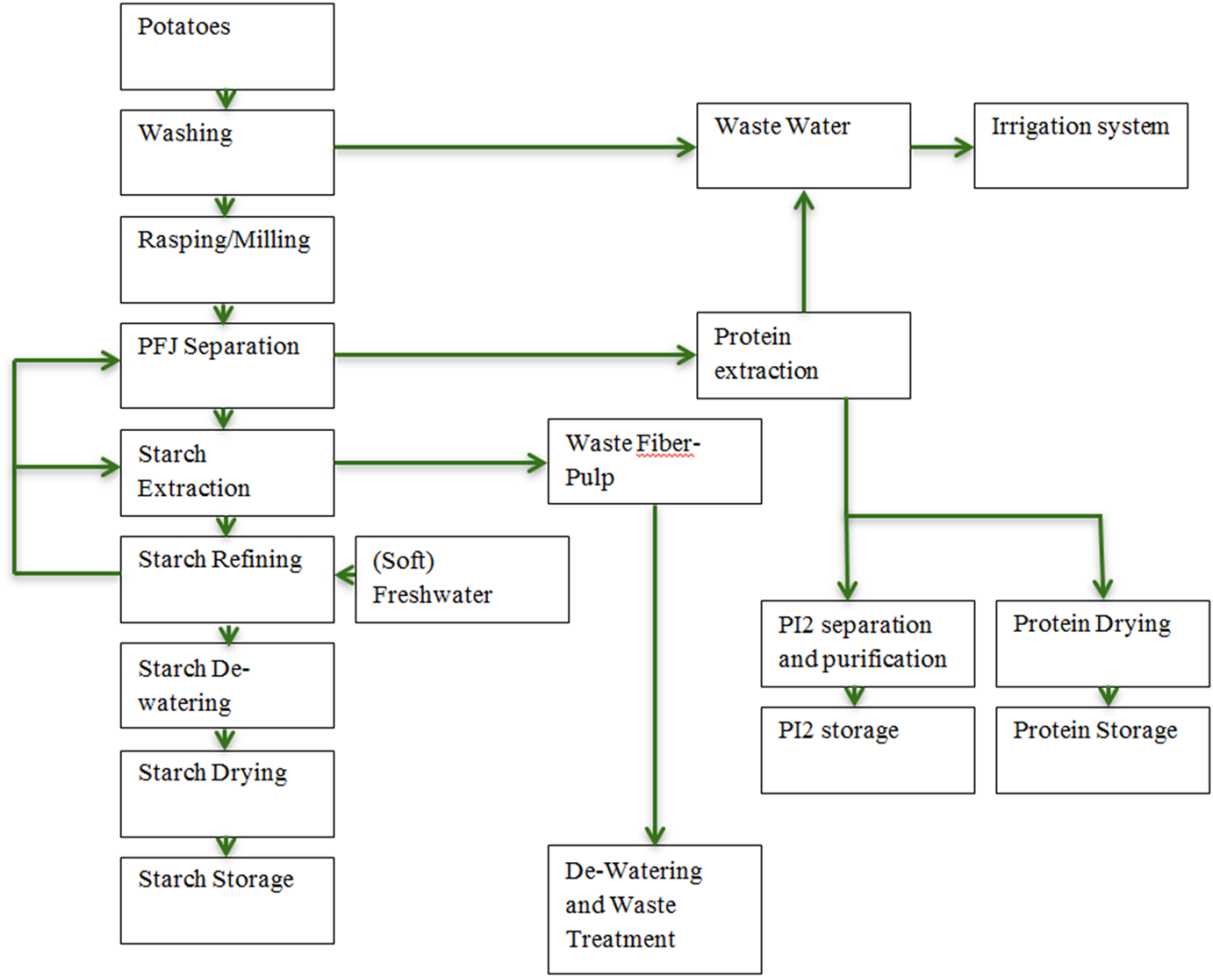
Process flow for proposed potato bio-refinery [298]

As mentioned earlier, 19% of a potato’s composition by weight is starch [136], but they also contain proteins; roughly 2% by weight, as well as other higher value components such as protease inhibitors found within the potato protein; these have been shown to have an appetite suppressing effect on mammals [269, 297]. Figure 25 summarises the main steps in a theoretical bio-refinery system based on existing systems already utilised within the potato industry.

A summary of the potential steps is as follows:

1. Removal of dirt/sand
  Washing of the tubers with water to remove excess dirt and sand. This aids in maintaining operational integrity in the system by removing impurities that could damage fast moving machinery.
2. Tuber milling/rasping
  Milling of the tubers to open tissue cells allowing extraction of the starch. Optimisation of this step is required to achieve maximum tissue milling without negatively affecting later filtration steps. Sodium hydrogen sulphite is sometimes required at this point to prevent discoloration. Achievable throughput has been quoted to be in the range of 20–30 t/h [298].
3. Separation of potato fruit juice (PFJ)
  There are a variety of systems described in the literature for the separation of the protein rich potato fruit juice, the majority utilise some form of centrifuge based decanter system to separate the liquid phase containing soluble proteins from the pulp containing the insoluble starch fraction. Because of the need for excess water to wash the pulp to ensure maximum protein recovery, wastewater from the starch purification step later in the supply line can be used, allowing for the reduction of water used within the system.
4. Fibre extraction
  Separation of the pulp resulting from the PFJ separation into fibre and starch using centrifugal sieves, jet extractors, or centrisieves. It has been quoted that optimised methods can yield a >95% yield of starch with a maximum throughput of 30 t/h [298].
5. Starch de-sanding, refining, de-watering, and drying
  De-sanding of the crude starch using hydrocyclones and several washes to remove protein and other soluble components (this water is recycled earlier in the process). Drying of the purified starch happens through a variety of methods; the one quoted in the literature involves use of rotating vacuum filters to reduce water content to 38% followed by flash drying to reach a final water content of 20%. Any aggregates produced in the drying step are fed back into the milling step.
6. Pulp de-watering
  Drying of the waste fibre-pulp produced to aid in waste treatment.
7. Protein recovery
  Protein recovery is an attractive addition to the existing starch extraction procedure, as it uses one of the current waste streams as the starting feedstock—for example, the PI2 protein discussed earlier. The wastewater from the starch process needs processing to obtain the protein; research on different precipitation methods is summarised in Table 5. The different methods for precipitation result in protein with different properties, so there is scope to tailor the precipitation method to suit the proposed protein use. There is also the issue of waste management; some of these precipitation methods use acid and therefore would complicate waste treatment procedures.

**Table 5:** Different protein yields and properties with regard to precipitation method [269]

Precipitation agent	Max protein yield (%)	Purification factor	Patatin (%)	PI 25–21 kDa (%)	PI 20–15 kDa (%)	PI <15 kDa (%)	HMW proteins (%)
Thermal/acid	90.2	0.74	37.9	0	20.2	31.3	10.7
Acid	64.7	1.26	11.1	9.9	15.3	17.4	46.4
FeCl_3_	75.2	6.24	21.7	18.7	23.2	34.3	2.0
MnCl_2_	16.8	1.52	20.4	0	30.9	44.2	4.6
Ethanol	55.2	3.79	37.7	8.0	22.4	26.5	5.4
(NH_4_)_2_SO_4_	98.8	2.99	31.1	7.6	23.7	26.3	11.3
  Summarised in the above table is the ratio of the various proteins in the extracted protein isolate. These are important to consider as they change the nature of the protein extracted. PFJ in its non-precipitated form has been quoted within the literature to contain 22.9–38% patatin, 45.6–56% protease inhibitors, and 9–23.7% higher molecular weight proteins; this variation is heavily dependent on the variety of potato extracted [269]. As can be seen, the ratio of proteins in the isolate is heavily dependent on the precipitation method, with more thermally labile proteins such as patatin being more prone to precipitation via heat, whereas thermally stable proteins such as the protease inhibitors are more sensitive to other precipitation methods. This shows scope for tailored precipitation methods depending on the intended application for the protein isolate.
  Another important factor to consider for use of extracted potato proteins as a food additive is the solubility in water. Once again, it is the protein precipitated using ammonium sulphate that performs particularly well here with solubility at pH 7.0 ranging from 78 to 89%; this indicates that the protein retains its conformational structure, proving that precipitation using ammonium sulphate is a soft technique (does not destroy/alter the protein).
8. Waste pulp and water
  The waste streams produced from this system require consideration. There are two main avenues of waste from the above-described process, one is the aqueous waste stream, and one is the solid pulp waste stream. The amount of waste water is reduced by recycling the fresh water used in the starch refining step in previous steps; this allows for the reduction of water used down to 0.4 m^3^/t of potatoes as quoted by Bergthaller et al. [298]. The unavoidable wastewater has the potential for re-use in the irrigation system used for growing the potatoes. The water stream will also be contaminated with the precipitation agent used to recover the protein isolate; this could potentially help in choosing the method of protein extraction; acidified water streams are obviously not desirable for crop irrigation, whereas use of ammonium sulphate (which is commonly used as a fertilizer) is more desirable. The waste fibrous pulp, however, requires further research into its potential valorisation routes.
9. Testing for glycoalkaloids
  Testing the concentration of glycoalkaloids within the extracted protein is very important if the intention is to produce it as a food additive. Alkaloids are frequently toxic at relatively low doses, yet can still display potentially useful characteristics in pharmacology if the dosage is low enough—e.g. atropine, which is the poison found in *Atropa Belladonna* (Deadly Nightshade) [299], is also on the World Health Organization’s list of essential medicines for a basic health system [300]. There have been many methods for determining glycoalkaloids reported in the literature including gas chromatography, thin layer chromatography, enzyme-linked immunosorbent assay, capillary electrophoresis, and MALDI-TOF MS. But the one method most used is HPLC [301]. Below is a brief summary of a typical HPLC assay for testing glycoalkaloid content in either the extracted starch or protein. Potato glycoalkaloids can be extracted using dilute acetic acid and sodium sulphite, the powdered protein is homogenised in this solution for 2 min, and the solid residue removed via centrifugation. The solution is then purified using solid phase extraction; HPLC analysis can then be carried out and using external standards, the concentration of both α-solanine and α-chaconine can be obtained [301].

### Citrus Fruits

Another significant case study in the use of a combined biorefinery is on citrus fruits, which includes oranges, lemons, limes, grapefruits, and tangerines. In 2013–2014 the major citrus producing countries, such as Brazil, China, India, US, EU-27, Mexico, Egypt, Turkey, and South Africa, produced around 140 million MT of these fruits, of which 60% were oranges [137]. Worldwide figures estimate that over 30% of citrus fruit produced (40% in the case of oranges) is processed by the food industry each year, as opposed to going directly to retail. This processing, including juicing and canning, generates large quantities of citrus peel waste (19 million tonnes annually). On top of this, and even though the harvesting season is fixed in specific locations worldwide [302], citrus fruits are grown and harvested around the globe on either side of the equator throughout the year, ensuring a constant supply of citrus peel waste that has the potential to be used as a bio-refinery raw material for valorisation purposes.

Citrus peel waste accounts for 50% of the whole fruit [303] and contains up to 80% water [304, 305]. It has been recognised as an interesting source of dietary fibre, natural antioxidants, food colorants, and flavours and is of particular interest given the variety of compounds it contains. Major components of dry citrus peel waste are cellulose (up to 37%), pectin (up to 23%), sugars (up to 23%, including glucose, fructose, sucrose, and xylose; dry weight basis), and up to 11% hemicellulose [304]. Other components also present in dry citrus peel include lignin (up to 10%) [305], flavonoids (up to 4.5%), and up to 4% of essential oil [306], mainly composed of d-limonene and often referred as citrus essential oil.

Amongst these compounds, d-limonene and pectin are the most attractive for industrial production, but the recovery of other compounds such as flavonoids and sugars are nowadays gaining importance for the reasons outlined previously. The production of d-limonene from waste citrus peel normally takes place after the essential oil extraction by traditional methods like cold pressing or steam distillation once the juicing process has finished [303, 307, 308]. The two different processes yield respectively a high purity/food grade and a technical grade of d-limonene as the latest involves the use of lime as a dewatering agent [305], hence limiting the final cellulosic-based end product to toxic cattle feed supplement [309]. On the other hand, pectin is traditionally extracted by acidic hydrolysis once the juice extraction process has concluded, generating quantities of acidic waste water [310, 311]. Still, this is not generally a by-product of the juice production industry as the inclusion of these extraction steps highly depends on the level capital available for investment, the required pay-back period and the juicing equipment used [312]. Moreover, the several washing and purification stages also involved make pectin production a wasteful and polluting process (particularly if the precipitation of pectin uses aluminium salts).

These conventional methods show some disadvantages related to high energy costs and long extraction times, and, according to the literature, the overall juicing process is becoming unprofitable and ineffective and would benefit from further improvements as current citrus waste processing is based upon processing technology that is at least 70 years old [309]. Scientists have, therefore, been developing greener and more efficient methodologies to be used in alternative biorefinery concepts to (1) increase production efficiency and (2) contribute to environmental preservation.

Clark et al. have recently developed a new biorefinery concept to combine all these extraction methods in an integrated process to be applied at industrial scale with the intention to bring together various new technologies [313]. As mentioned previously, conventional techniques tend to require hazardous solvents or additives to carry out a successful extraction of products from citrus peel waste. Thus, a methodology to produce a wide range of marketable products and relying on green solvents and techniques and able to cope with wet feedstock would be advantageous. Here lies the advantage of the microwave protocol compared to other techniques. Recently, microwave technology has gained increased industrial interest in the food sector since it (1) can be applied directly to the desired biomass without any need of solvent or pre-treatment (i.e. drying); (2) allows a rapid and homogeneous heating; and (3) is adaptable for continuous processes and easily scalable [314]. This is a key factor for future industrial scale applications given the importance of citrus peel drying costs. The scalability of this technology has been proved in different studies and has shown important advantages over traditional methods [315]. These features allow microwave technology be applied to different systems in order to obtain more flexible processes, leading to lower energy consumption and environmental impact [316].

Microwave heating has been previously used as a faster, more efficient and cost effective alternative for the extraction of higher quality d-limonene [317, 318] and pectin [319–321] from citrus peel waste. However, most of these individual methods still involve the use of additives and/or additional pre-treatment steps.

The methodology developed by Clark et al. [313] shows that it is possible to treat fresh waste orange peel (WOP) through three steps without involving additional chemicals for subsequent extraction: (1) d-limonene using microwave energy, (2) sugars and flavonoids after washing the residual citrus peel with hot ethanol, and (3) pectin following a pressurised microwave extraction under acid-free conditions, finally to end up with a cellulose-based solid material, and resulting in an innovative zero-waste biorefinery concept.

### Bio-Boards

Coming from the other angle, the final case study presented gives an example of where the differently processed parts of several biowastes are brought together to make a finished product. MDF and particle boards are very highly used in furniture construction—the UK is estimated to produce over three million tonnes annually [137]. The process typically involves using chipped wood or other lingo-cellulosic substance being pressed or moulded into panels and bound together in place with urea–formaldehyde as a binder. The current issues associated with this process are:

1. Competition for virgin wood for other purposes coupled with the need to preserve forests.
2. Petroleum is the current source for urea–formaldehyde.
3. Formaldehyde is currently listed as a “probable carcinogen” and is continually emitted from the boards through their lifespan [322].

**Fig. 26:**
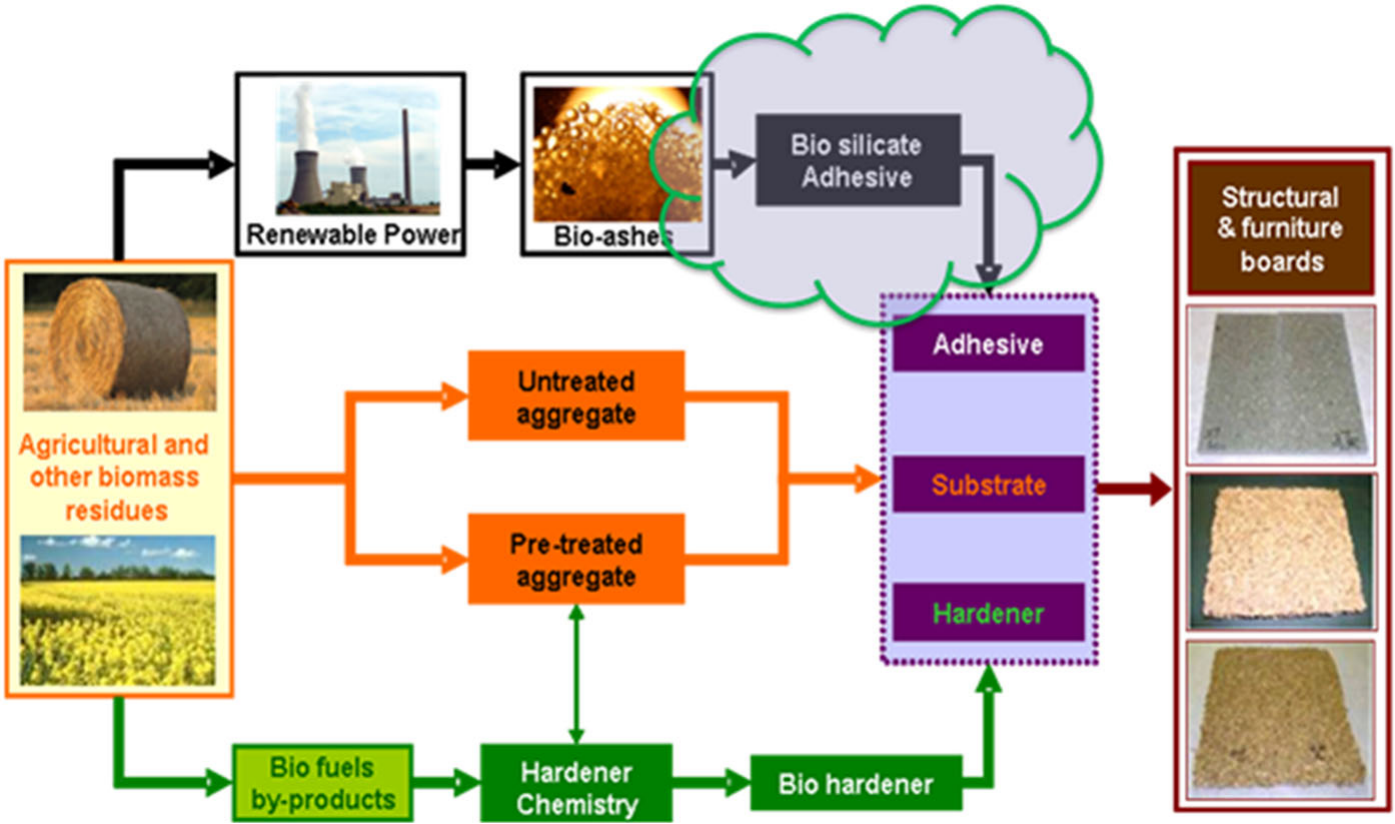
The schematic overview of the production of bio-boards [330]

A joint project between the Universities of Bangor and York, as well as several private companies investigated using waste wheat straw as an alternative raw material for particleboards and manufacturing them in a greener manner. First, the wheat straw is de-waxed, with one portion of the straw going for biomass burning for renewable energy and the other directly to make the boards. Potential applications for the waxes include a variety of coatings, plasticisers, and cosmetic bases amongst others [323–326]. Meanwhile, the ashes from the biomass burning then have the silicates extracted with sodium- or potassium-based alkali solutions [327, 328]. The resulting silica solutions can then subsequently be used as the binder for the wheat straw alongside either whey protein or glycerol with the resulting boards pressed in the same manner as conventional particleboard [329]. The resulting boards have been assessed to meet all EN criteria and now produced at scale [330]. With the wax extracted for various purposes and the wheat straw used either directly in the board itself or via the power station as ash, this strategy represents full usage of wheat straw in a biorefinery concept. In addition, it demonstrates the “big picture” of biorefineries by integrating products resulting from the biorefinery of other biowastes, e.g. whey protein from dairy effluent or glycerol from biodiesel/soap production. The process is summarised in Fig. 26.

## Concluding Remarks

Overall, the prospects for using waste biomass from existing industrial processes—notably food production—as the source for many of our materials is becoming increasingly close to realisation. Sugars and other carbohydrates are arguably the easiest and most versatile of the three main biological macromolecules due to their relative similarity in structure (allowing for more broad-stroke techniques) and the fact that they are already used a feedstock for bacterial cultures for many existing fermentation processes. The range of molecules produced includes platform molecules for the production of materials, monomers capable of polymerisation directly to materials and solvents used for the processing stages.

Lipids present less versatility, but again their similarity in structure allows for the application of a number of broad-stroke techniques and their hydrophobicity makes them useful as replacements for a number of crude oil applications such as surfactants, lubricants, waxes, and polymers.

Proteins represent a class of materials in their own right due to the structures and functions they provide in biological systems. However, because of this they are by far the most diverse of the three macromolecules and are arguably the hardest to develop a processing method for due to the lack of broad-brush techniques available to carbohydrates and lipids. Extraction, in particular, is a major barrier to overcome in order to achieve processing to green materials. However, there are a number of promising developments in this field and their diversity represents significant promise for versatility in the range of materials that could arise from the biorefinery concept.

The case studies presented highlight the ability to utilise all parts of biomass sequentially in the biorefinery concept as well potential for different fractions to come together when producing new materials. They also illustrate that the minor components of biomass, such as vitamins and minerals, are also important when considering refining biomass, as they also represent the potential to be feedstocks for niche applications.

## Footnotes

1 High value in this context means additional to economic value created from liquid biofuels (bioenergy) within the context of a biorefinery.
